# Iron homeostasis and ferroptosis in muscle diseases and disorders: mechanisms and therapeutic prospects

**DOI:** 10.1038/s41413-024-00398-6

**Published:** 2025-02-25

**Authors:** Qin Ru, Yusheng Li, Xi Zhang, Lin Chen, Yuxiang Wu, Junxia Min, Fudi Wang

**Affiliations:** 1 Institute of Intelligent Sport and Proactive Health, Department of Health and Physical Education, Jianghan University, Wuhan, China; 2 Department of Orthopedics, Xiangya Hospital, Central South University, Changsha, China; 3 National Clinical Research Center for Geriatric Disorders, Xiangya Hospital, Central South University, Changsha, China; 4 The First Affiliated Hospital, Institute of Translational Medicine, Zhejiang University School of Medicine, Hangzhou, China; 5 The Second Affiliated Hospital, School of Public Health, State Key Laboratory of Experimental Hematology, Zhejiang University School of Medicine, Hangzhou, China

**Keywords:** Metabolism, Diseases, Pathogenesis

## Abstract

The muscular system plays a critical role in the human body by governing skeletal movement, cardiovascular function, and the activities of digestive organs. Additionally, muscle tissues serve an endocrine function by secreting myogenic cytokines, thereby regulating metabolism throughout the entire body. Maintaining muscle function requires iron homeostasis. Recent studies suggest that disruptions in iron metabolism and ferroptosis, a form of iron-dependent cell death, are essential contributors to the progression of a wide range of muscle diseases and disorders, including sarcopenia, cardiomyopathy, and amyotrophic lateral sclerosis. Thus, a comprehensive overview of the mechanisms regulating iron metabolism and ferroptosis in these conditions is crucial for identifying potential therapeutic targets and developing new strategies for disease treatment and/or prevention. This review aims to summarize recent advances in understanding the molecular mechanisms underlying ferroptosis in the context of muscle injury, as well as associated muscle diseases and disorders. Moreover, we discuss potential targets within the ferroptosis pathway and possible strategies for managing muscle disorders. Finally, we shed new light on current limitations and future prospects for therapeutic interventions targeting ferroptosis.

## Introduction

The muscular system serves as the body’s main structure for both movement and stability. The human body contains more than 600 named muscles, comprising nearly half of the total body weight. Virtually all physical movement requires muscles, which can be broadly categorized into two groups: striated muscles, including skeletal muscle and cardiac muscle; and non-striated muscles, such as smooth muscles in the vascular, respiratory, and gastrointestinal systems.^1^ In addition to their roles in physical movement and stability, muscles also function as endocrine organs that affect systemic metabolism by releasing a wide range of cytokines.^2,3^ Muscle diseases and disorders, such as sarcopenia, amyotrophic lateral sclerosis (ALS), and cardiomyopathy drastically reduce the quality of life and can even be life-threatening. Moreover, the global burden associated with muscle diseases and disorders is currently increasing.^4,5^ Therefore, understanding the pathogenic mechanisms underlying muscle diseases and disorders is of high clinical significance, and the development of targeted therapeutic strategies is an urgent unmet medical need. As the most abundant essential metal element in the human body, iron is involved in oxygen transport, storage, and energy metabolism, which is necessary to maintain the normal physiological function in muscles. Therefore, the in-depth study of iron metabolism in muscle cells and iron deficiency/overload on muscle function may point out a new direction for the prevention and treatment of muscle diseases and disorders.^6–8^

Ferroptosis is a cellular process dependent upon excess iron, reactive oxygen species (ROS), and phospholipids (PLs) that contain polyunsaturated fatty acid chains (PUFAs).^9,10^ Ferroptosis can be initiated by two primary pathways, namely the exogenous (transporter-dependent) pathway and the endogenous (enzyme-regulated) pathway.^11,12^ The exogenous pathway is triggered by the inhibition of transporters at the cell surface, such as the cystine/glutamate transporter (also known as system Xc^−^), or by the activation of iron transporters. In contrast, the endogenous pathway is primarily initiated by blocking the expression and/or activity of intracellular antioxidant enzymes, including the glutathione (GSH)/glutathione peroxidase 4 (GPX4) pathway^13–15^ and the ferroptosis suppressor protein 1 (FSP1)/coenzyme Q10 (CoQ10) pathway,^16^ and by activating enzymes involved in fatty acid metabolism such as acyl-CoA synthetase long-chain family member 4 (ACSL4).^17–21^ Key features in the induction of ferroptosis include increased iron accumulation, the generation of free radicals, an imbalance between oxidative and antioxidant systems, as well as lipid peroxidation.^22^

Although the field of ferroptosis research remains in its infancy, the volume of studies on this topic has surged exponentially since its initial characterization by Dixon et al. just over a decade ago. A growing number of evidence indicates that ferroptosis plays a significant role in the onset and progression of various muscle diseases and disorders.^6,23–25^ For example, changes in multiple metabolic pathways associated with ferroptosis have been implicated in skeletal muscle atrophy, cardiac injury, and motor neuron loss.^22,26,27^ Therefore, targeting ferroptosis may provide a promising new therapeutic strategy for treating and/or preventing these conditions. In this review, we offer an in-depth analysis of the mechanisms and processes that regulate ferroptosis, while also summarizing recent advancements with the role of ferroptosis in various muscle diseases and disorders. In addition, we present practical and experimental insights into the diagnosis and treatment of these diseases and disorders and investigate emerging putative targets and intervention strategies for the development of novel clinical therapies aimed at modulating ferroptosis.

## Iron homeostasis and ferroptosis in the muscular system

**Fig. 1:**
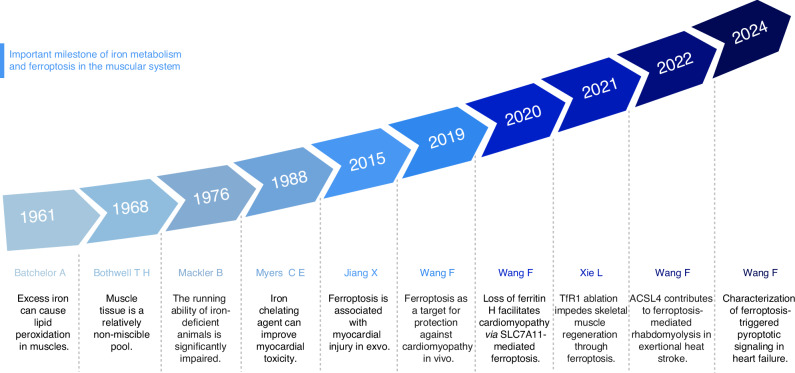
The important milestone of iron metabolism and ferroptosis in the muscular system. The figure presents a timeline highlighting significant milestones in the study of iron metabolism and ferroptosis in the muscular system from 1961 to 2024, including the influence of iron metabolism on muscle function, and the regulation of antioxidant system and lipid metabolism on muscle ferroptosis. This figure was created with BioRender (https://biorender.com/)

The content and distribution of iron in the human body are maintained in a relatively stable state, a condition referred to as “iron homeostasis”, which is fundamental for sustaining normal physiological functions.^28,29^ As early as 1968, Bothwell et al. found that although the concentration of iron stored in muscle is much lower than that in liver, the total amount of iron stored in muscle is at least equal to the amount of iron stored in the liver due to the large muscle mass. The iron stored in the muscle is a relatively immiscible pool that responds little to sharp changes in the iron environment^30^ (Fig. 1). Iron deficiency or iron overload can cause abnormal muscle cell metabolism, leading to diseases and disorders.^31–33^ When the supply of iron to cells exceeds their physiological requirements, resulting in the accumulation of excessive iron in cells and causing lipid peroxidation, this phenomenon may induce ferroptosis,^34^ which has been reported in association with sarcopenia and cardiomyopathy due to multiple causes.^23,35–39^

**Fig. 2:**
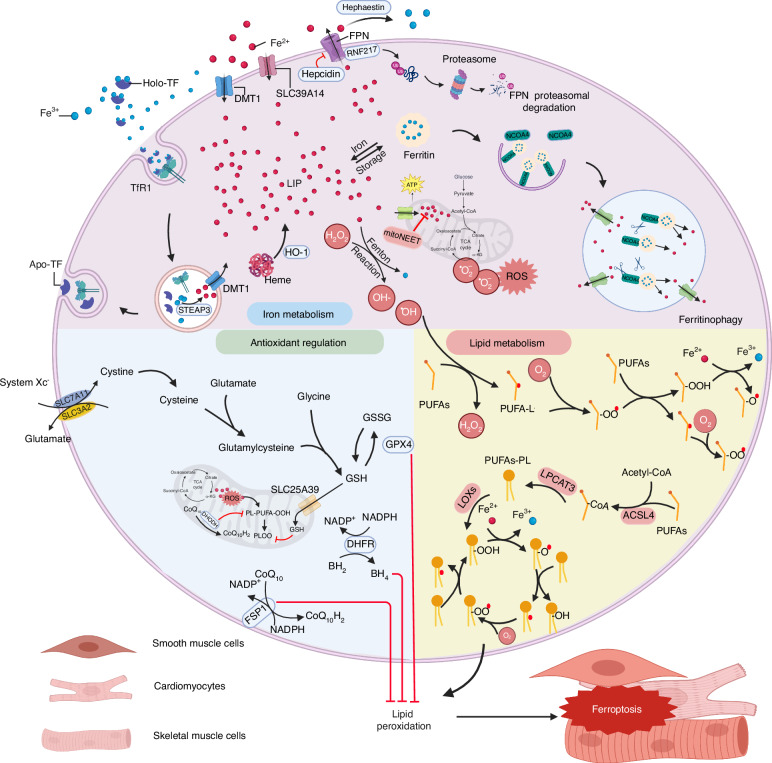
Overview the mechanisms underlying ferroptosis in skeletal, cardiac, and smooth muscle. Iron overload is one of the principal driving factors of ferroptosis. Many aspects of iron metabolism such as the iron absorption, storage, and utilization are involved in regulating ferroptosis. When cellular iron is sufficient, transferrin-bound iron is reduced in order to limit excess iron accumulation. However, under iron-overload conditions, the uptake of non-transferrin-bound iron increases, facilitated by metal transporter proteins such as SLC39A14. In general, excess iron is stored in ferritin-bound substances or in the labile iron pool (LIP), which can increase due to a metabolic imbalance. Iron in the LIP participates in the production of reactive oxygen species (ROS) via the Fenton reaction. NCOA4 is a cargo receptor that binds ferritin, transporting it to autolysosomes, leading to the release of free iron. The classic intracellular pathway for inhibiting ferroptosis involves the uptake of cystine through the cystine-glutamate transporter (system Xc^−^), which results in the biosynthesis of GSH. GPX4 reduces phospholipid hydroperoxide, using GSH as a cofactor. FSP1 also reduces CoQ10 and inhibits the oxidation of phospholipids. In addition, activation of ACSL4, LPCAT3, and LOXs plays a role in ferroptosis by promoting lipid peroxidation. This figure was created with BioRender (https://biorender.com/). Abbreviations: ACSL4 acyl-CoA synthetase long-chain family member 4, DMT1 divalent metal transporter1, FPN ferroportin, FSP1 ferroptosis suppressor protein 1, GPX4 glutathione peroxidase 4, GSH glutathione, HO-1 heme oxygenase 1, LIP labile iron pool, LOXs lipoxygenases, LPCAT3 lysophospholipid acyltransferase 3, NCOA4 nuclear receptor coactivator 4, PUFA polyunsaturated fatty acid, RNS reactive nitrogen species, ROS reactive oxygen species, SLC39A14 (ZIP14) Zrt and IRT-like protein 14, STEAP3 six transmembrane epithelial antigen of prostate 3, TF transferrin, TfR1 transferrin receptor protein 1

The most prominent morphological features in ferroptosis include increased mitochondrial density, the reduction or complete loss of mitochondrial cristae, and rupture of the outer mitochondrial membrane.^40^ Biochemically, ferroptosis is characterized primarily by iron accumulation and increased lipid peroxidation; thus, progress in three key areas—antioxidant regulation, lipid metabolism, and iron metabolism—has significantly contributed to our understanding of ferroptosis^41^ (Fig. 2). The identification of these metabolic pathways has also deepened our insights into the role of ferroptosis in muscle diseases and disorders. Compared to other tissues, muscle tissue requires more energy to maintain posture and movement, therefore the muscular system has a higher number of intracellular mitochondria.^42,43^ Mitochondria host a wide range of key metabolic processes (such as the tricarboxylic acid cycle) and are a major source of ROS, which makes muscle cells more sensitive to ferroptosis.

### Iron metabolism and ferroptosis in the muscular system

Iron is an important trace element that supports the life of organisms. Iron metabolism refers to the process in which iron is absorbed, transported, distributed, stored, utilized, transformed, and excreted in organisms. The metabolic process of iron has a perfect control system to maintain a relative balance between iron absorption and excretion.^44^ The importance of iron to life and health is far more complex and diverse than humans have predicted, at present, the research methods of iron in living organisms involve many disciplines. Professor Wang has proposed a new interdisciplinary science focused on iron, called “Ferrology”,^45^ and the emergence of this iron-focused discipline will greatly improve our understanding of the role of iron homeostasis in maintaining and promoting human health, while helping to develop new disease treatment strategies targeting ferroptosis.

The small intestine serves as the major organ for dietary iron absorption, with an average of 1~2 mg of iron absorbed daily by intestinal enterocytes into the systemic iron pool.^46^ Circulating iron is taken up by muscle cells and plays a vital role in their metabolism. In myocardial cells, for example, iron serves as a cofactor for enzymes involved in the tricarboxylic acid cycle, while iron-sulfur protein is critical molecule of the mitochondrial respiratory chain. Iron deficiency can cause mitochondrial dysfunction and heart failure.^47,48^ In skeletal muscle, myocardial muscle and smooth muscle, iron is an essential component of myoglobin, which is involved in the production of mitochondrial energy by transporting and storing oxygen within muscle cells.^49^

#### Iron homeostasis is required for normal muscle function

Under normal conditions, iron homeostasis is maintained by a balance between the cellular intake, utilization, and efflux of iron. Systemic iron is bound primarily to the protein transferrin (TF), which contains two high-affinity sites for ferric iron binding.^50^ And cellular iron uptake is mediated by TF and its receptor transferrin receptor protein 1 (TfR1).^51,52^ Mice lacking TfR1 develop fatal cardiomyopathy with failed oxidative phosphorylation and impaired mitophagy, which can be mitigated by iron supplementation that overwhelms the capacity of serum TF to bind iron.^53^ Skeletal muscle-specific *Tfr1* knockout mice exhibit severe iron deficiency, are smaller than their wild-type littermates due to growth retardation, and typically die or require euthanasia by the second postnatal week.^54^ Loss of skeletal muscle *Tfr1* decreases the expression of iron-containing electron transport chain (ETC) complexes, resulting in impaired mitochondrial respiration and reduced energy production in skeletal muscle, with subsequent growth impairment, all of which are reversed by high doses of iron dextran.^54^ Interestingly, cardiac levels of non-transferrin-bound iron (NTBI) in homozygous global *Slc39a14* (also known as Zrt and IRT-like protein 14, or ZIP14) knockout mice were ~60% higher than those in wild-type mice, with intermediate levels measured in heterozygous *Slc39a14* knockout mice. This suggests that SLC39A14 plays a crucial role in mediating NTBI uptake in cardiomyocytes, with a gene dose-dependent effect on cardiac iron accumulation.^55^

Once inside the cell, some of the ferrous iron atoms are oxidized to the ferric iron state by cytoplasmic ferritin and are stably bound to ferritin for either enzymatic reactions or storage for later use; the remaining ferrous iron atoms reside in the so-called labile iron pool.^56,57^ Nuclear receptor coactivator 4 (NCOA4) mediates the selective autophagic degradation of ferritin through a process known as ferritinophagy,^58^ thereby playing a critical role in both intracellular and systemic iron homeostasis.^58^ In mice with sepsis, myocardial levels of ferroptosis markers—such as prostaglandin-endoperoxide synthase 2 (PTGS2), malondialdehyde (MDA), and lipid ROS—are significantly elevated. These mice also exhibit increased NCOA4 expression and intracellular Fe^2+^ levels, alongside decreased ferritin levels,^59^ suggesting that ferritinophagy-mediated ferroptosis is a key mechanism underlying sepsis-induced cardiac injury. Additionally, cytoplasmic Fe^2+^ was shown to enhance the expression of siderofexin in the mitochondrial membrane, which facilitates the transport of cytosolic Fe^2+^ into the mitochondria, thereby promoting mitochondrial ROS production and ferroptosis.^59^ The selective loss of *Fth* (ferritin heavy chain) in cardiomyocytes results in decreased cardiac iron content and increased oxidative stress in the heart, leading to mild cardiac injury during aging. Furthermore, the absence of cardiac FTH heightens the susceptibility of cardiac tissue to iron overload-induced damage, and cardiac-specific *Fth* knockout mice fed with high-iron diet demonstrated severe heart damage and hypertrophic cardiomyopathy.^37^

The efflux of iron is mediated by ferroportin (FPN), with hepcidin inhibiting FPN through E3 ubiquitin-protein ligase ring finger protein 217 (RNF217)-mediated ubiquitination, thereby regulating systemic iron absorption and iron recycling.^60–62^ However, the role of FPN in maintaining iron homeostasis within the myocardium remains controversial. Lakhal-Littleton et al. demonstrated that cardiac ferritin and iron levels were markedly elevated in myosin heavy chain 6 (*Myh6)-Cre*‒induced cardiomyocyte-specific *Fpn* knockout mice (*Fpn*^*Myh6/ Myh6*^ mice), which exhibited severely compromised cardiac function.^63^ These mice also showed increased mortality in comparison to corresponding controls, with a median survival of only 5.5 months,^63^ suggesting that cardiac FPN is essential for intracellular iron homeostasis. In contrast, Wang and colleagues reported that muscle creatine kinase (*MCK)-Cre* cardiomyocyte-specific *Fpn*-deficient mice (*Fpn*^*MCK/ MCK*^ mice) did not develop any significant phenotype, with no substantial iron accumulation in myocardial tissue, even when fed an iron-rich diet.^64^ These divergent phenotypes may be attributed to the timing of the Cre enzyme expression in the respective models.^64^ *Myh6-Cre* is expressed earlier in the embryonic heart than *MCK-Cre*, therefore, the earlier loss of cardiac FPN in *Fpn*^*Myh6/ Myh6*^ mice may contribute to their more severe phenotype.

Notably, cellular iron accumulation increases during aging, and age-related iron homeostasis is often associated with a variety of age-related diseases, including musculoskeletal system diseases.^65–67^ The chronic inflammatory state of the elderly population increases ferritin levels, which makes ferritin a potential biomarker of age-related inflammation.^68,69^ In general, serum iron and soluble TfR1 levels decrease with age, while ferritin levels increase with age.^70^ For example, cross-sectional studies of American populations have shown that high ferritin levels are associated with shorter telomeres in people aged 65 years and older.^71^ The skeletal muscle of elderly rats has higher iron levels, lower TfR1 and FPN levels, and increased expression of non-heme iron transporter SLC39A14, resulting in increased accumulation of unstable iron.^23^ Under normal circumstances, ferritin can be broken down by autophagy, releasing iron for use in cellular processes. However, autophagy in skeletal muscle is impaired with age, which may lead to poor recycling of ferritin from muscle in older animals.^67,72^ Overall, iron homeostasis appears to be intuitively linked to human aging, with age-related iron homeostasis contributing to a variety of human diseases including osteoporosis and sarcopenia.

#### Iron overload-associated muscle dysfunction

Under certain pathological conditions, excessive iron accumulation in the muscles has been observed, even with a normal diet. For instance, exogenous ferric citrate can induce cell death of myoblasts and impair their differentiation from myoblasts to myotubes, and skeletal muscle atrophy has been found in aged mice, accompanied by iron accumulation.^51,73^ In transgenic rats (an animal model of ALS) carrying the *G93A hmSOD1* (superoxide dismutase 1) gene, muscle iron content was shown to increase with disease progression.^74^ Iron overload may also lead to thalassemia-associated cardiomyopathy.^75^ Iron chelation therapy, first introduced in the 1970s to manage adverse effects of iron overload in patients with thalassemia-associated cardiomyopathy, has been globally recognized as the most effective treatment for this condition.^76,77^

#### Iron deficiency-associated muscle dysfunction

Iron is essential for cellular processes such as energy metabolism, nucleotide synthesis, and many enzymatic reactions.^37,78^ Iron deficiency impairs aerobic glycolytic capacity, reduces myoblasts proliferation, and induces apoptosis in myocytes.^79^ Mechanistically, iron deficiency induced by the iron-chelating agent deferiprone (DFP) increases the levels of B-cell lymphoma 2 (BCL2)-interacting protein 3, causing a loss of skeletal muscle mitochondrial proteins and consequent reduction in respiratory capacity.^80^ Bloodletting and three weeks on a low-iron diet in mice reduces skeletal muscle iron stores, significantly diminishing activity of respiratory chain complex I and muscle endurance.^81^

A large population cohort study revealed that iron deficiency is associated with reduced muscle mass.^79^ Moreover, iron deficiency is common in patients with chronic heart failure and is associated with disease severity, with iron deficiency serving as an independent predictor of mortality.^82^ Low cardiac iron levels have been implicated in the progression of heart failure in experimental models, with patients suffering from advanced heart failure with reduced ejection fraction exhibiting lower iron concentrations.^83^ Intravenous administration of carboxymaltose iron mitigates symptoms and enhances the quality of life in heart failure patients.^84^ Iron deficiency was also shown to impair mitochondrial adenosine triphosphate (ATP) production in cardiomyocytes, directly reducing their contractility and relaxation; restoring intracellular iron levels reverses these effects.^85,86^ The sodium-glucose cotransporter 2 inhibitor dapagliflozin has been reported to alleviate cytoplasmic iron deficiency in patients with heart failure by modulating the activity of proteins involved in iron homeostasis.^87,88^

#### Iron imbalance induces ferroptosis in the muscular system

In animal models, alterations in various aspects of iron metabolism—such as increased iron absorption, reduced iron storage, and limited iron efflux—can result in elevated iron accumulation. Global *Trf* knockout mice typically die within one day after birth; however, in hepatocellular conditional *Trf*-deficient mice, serum levels of TF-bound iron (TBI) are significantly reduced, leading to NTBI overload in various tissues such as the heart.^89^ In the absence of TF, NTBI can be transferred to cells primarily by SLC39A14, contributing to tissue iron accumulation.^89^ Notably, both dietary supplementation with apo-TF, which alleviates iron overload, and the loss of SLC39A14 substantially reduce high serum NTBI levels and iron deposition in various tissues, thereby inhibiting cellular ferroptosis.^89^ Excess iron promotes subsequent lipid peroxidation, triggering ferroptosis through at least two mechanisms: i) the generation of ROS via the iron-dependent Fenton reaction, and ii) the activation of iron-containing enzymes such as lipoxygenases (LOXs).^90,91^ Autophagic degradation of ferritin exacerbates ferroptosis by increasing intercellular iron levels,^92,93^ while the membrane glycoprotein prominin2 promotes the formation of ferritin-containing multivesicular bodies and exosomes to export iron, thus suppressing ferroptosis.^94^ Several mitochondrial proteins, including cysteine desulfurase 1 (NFS1) and iron-sulfur cluster assembly scaffold protein IscU, negatively regulate ferroptosis by reducing mitochondrial and intracellular iron through their roles in the biosynthesis of iron-sulfur clusters.^95–97^ These findings collectively highlight the complex regulation of iron homeostasis and its critical role in ferroptosis.

#### Iron homeostasis in satellite cell self-renewal, differentiation, and fusion

Adult skeletal muscle in mammals is stable tissue under normal circumstances, but has a remarkable ability to repair after muscle injury. Skeletal muscle regeneration is a highly regulated process involving the activation of various cellular and molecular reactions, and is a dynamic balance process between satellite cells as stem cells and their microenvironment. Satellite cells, as progenitors of skeletal muscle, are a class of undifferentiated monocytes located in the space between the basal membrane of muscle fibers and the cell membrane of muscle. When the muscle cells are damaged by external stimulation, they are activated to form myoblasts, which have the ability to proliferate and differentiate, repair the damaged muscle fibers, and fuse with the original muscle cells to rebuild muscle fibers and regenerate skeletal muscle.^98^ Self-renewing proliferation and differentiation of satellite cells not only maintains the stem cell population, but also provides a large number of myoblasts, which fuse and lead to the formation of new muscle fibers and the reconstruction of skeletal muscles.^99^

Iron is critical to cell metabolism and energy production, and its availability affects the proliferation and self-renewal of satellite cells. The imbalance of iron metabolism may damage the delicate balance needed for the maintenance of satellite cells, potentially damaging the ability of them to effectively regenerate muscle tissue. *Tfr1* loss in myosatellite cells led to unstable iron accumulation, reduced GPX4 and nuclear factor erythroid 2-related factor 2 (Nrf2) levels, and lipid peroxidation in myosatellite cells, resulting in irreversible reduction of myosatellite cells and reduced proliferation and differentiation capabilities, leading to impaired skeletal muscle regeneration. Single-cell sequencing revealed a significant increase in genes associated with iron transport and oxidative stress in skeletal muscle satellite cells of patients with peripheral artery disease (PAD), while GPX was significantly reduced, and histologically confirmed the presence of iron deposits in skeletal muscle of patients with PAD.^100^ Iron overload caused increased oxidative stress and decreased expression of satellite cell markers in skeletal muscle. Cardiotoxin (CTX) could cause muscle injury and induce skeletal muscle regeneration. Mice with iron overload showed delayed muscle regeneration, reduced size of regenerated muscle fibers, decreased expression of myoblast differentiation markers, and decreased phosphorylation of mitogen-activated protein kinase (MAPK) signaling pathway after CTX-induced muscle injury.^101^ In addition, iron deficiency also reduces myoglobin expression and mitochondrial oxygen-consuming capacity, thereby reducing myoblast proliferation.^79^ Therefore, the imbalance of iron metabolism may destroy the ability of satellite cell proliferation, which may damage their ability to regenerate muscle tissue.

When muscle fibers are damaged, satellite cells are activated and begin to divide asymmetrically, and some daughter cells enter the cell cycle to proliferate and undergo myogenic differentiation. The regulation of iron on key signaling pathways and molecules in muscle satellite cells is crucial to the differentiation of satellite cells in mature muscle fibers. Fe^2+^ accumulation can decrease the differentiation capacity of C2C12 cells, and iron overload inhibits the differentiation of C2C12 myoblasts in vitro,^101^ while taurine accumulation can significantly increase the level of GSH, decrease the expression of heme oxygenase-1 (HO-1), decrease the level of Fe^2+^, thereby rejuvenating impaired myogenic differentiation.^102^ Reduced nicotinamide adenine dinucleotide (NADH) dehydrogenase (ubiquinone) iron-sulfur protein 8 (Ndufs8) expression in resting muscle satellite cells is quite low, but significantly increased in activated muscle satellite cells. In addition, the expression of Ndufs8 in skeletal muscle of old mice is also significantly reduced compared with that of young mice.^103^ Overexpression of Ndufs8 stimulates the myogenic capacity and metabolic changes of satellite cells, while inhibition of Ndufs8 weakens myogenic capacity and anti-apoptotic capacity, and the mechanism may be related to the regulation of intracellular NAD/NADH ratio and sirtuin (SIRT) activation to affect p53 acetylation.^103^

Furthermore, myoblasts formed by the differentiation of satellite cells need to fuse with existing muscle fibers to restore tissue integrity during muscle regeneration. The availability of ions may affect the efficiency of this fusion process. For example, the mechanosensitive, non-selective cationic channel Piezo1 increases in expression in differentiated myoblasts, and knockdown of *Piezo1* leads to a significant decrease in myoblast fusion and subsequently impedes the formation of myotubes, while overactivation of Piezo1 causes myoblast fusion.^104,105^ The uptake of iron in muscle formation is mainly the result of TfR-mediated endocytosis. Compared with resting satellite cells, TfR expression is increased in exponentially growing myoblasts, and the maximum iron uptake rate in myotubes is significantly higher than that in myoblasts.^106^ These findings suggest that iron-dependent proteins and pathways may be involved in mediating myoblast fusion.

### Lipid metabolism and ferroptosis in the muscular system

#### Intracellular lipid oxidation

Polyunsaturated fatty acid-acyl-CoA (PUFA-CoA) catalyzes the addition of PUFAs to the sn-2 position of phospholipids by esterification, a process mediated by ACSL4; thus, decreasing ACSL4 expression can reduce this process.^107^ ACSL3, another enzyme, converts monounsaturated fatty acids (MUFAs) into their acyl coenzyme esters, which then bind to membrane phospholipids, and this process may competitively inhibit PUFA peroxidation.^108^ The lipid oxidation process also requires the enzyme lysophosphatidylcholine acyltransferase 3 (LPCAT3) and is closely related to phospholipid remodeling, incorporating PUFA into phosphatidylethanolamines (Pes).^109,110^ LOXs, a family of iron-containing enzymes, directly oxidize PUFAs and PUFA-containing lipids within cell membranes and trigger lipid peroxidation by introducing hydroxyl groups (-OOH) into the fatty acid chain, with various LOXs such as arachidonate 12-lipoxygenase (ALOX12) mediating lipid peroxidation to produce hydroperoxide.^111,112^ Several membrane electron transfer proteins—particularly P450 oxidoreductase and nicotinamide adenine dinucleotide phosphate (NADPH) oxidases (NOXs)—also contribute to the production of ROS during lipid oxidation.^113,114^

#### Lipid peroxidation triggers ferroptosis in the muscular system

Although iron overload is an important cause of ferroptosis, it is not the only decisive factor. The second key factor in ferroptosis is the presence of readily oxidized PUFAs within cells and the accumulation of intracellular PUFAs increases the sensitivity of cells to ferroptosis inducers. Phospholipids are essential components of biological membranes; therefore, cell membranes are the main targets of oxidative damage in ferroptosis. The accumulation of lipid peroxides is a key feature of ferroptosis, ultimately driven by the peroxidation of specific membrane lipids. In ferroptosis, PUFAs—particularly arachidonic acid and adrenergic acid—are highly susceptible to peroxidation, leading to the destruction of the lipid bilayer and compromising membrane function. The biosynthesis and remodeling of PUFA-containing phospholipids in cell membranes require the enzymes ACSL4 and LPCAT3. Decreasing ACSL4 expression^107^ and knocking down LPCAT3 have been shown to protect against ferroptosis.^109^ Cells lacking ACSL4 exhibit higher levels of free oxidized PUFAs compared to esterified oxidized PUFAs, and inhibiting ACSL4 in wild-type cells protects against ferroptosis induced by the GPX4 inhibitor RAS-selective lethal small molecule 3 (RSL3).^18^ Thus, although system Xc^‒^ and GPX4 generally function to potently suppress ferroptosis, both *ACSL4* and *LPCAT3* are considered the first identified ferroptosis genes due to their role in promoting the incorporation of PUFAs into membrane lipids.^18^ In contrast, exogenous MUFAs reduce the sensitivity of plasma membrane lipids to cytotoxic oxidation, and ACSL3 catalyzes the conversion of exogenous MUFAs into fatty acyl-CoAs, thereby replacing PUFAs and inhibiting ferroptosis; thus, exogenous MUFAs and ACSL3 activity contribute to a ferroptosis-resistant cellular state.^108^

In skeletal muscle, ACSL4-mediated ferroptosis has been reported to exacerbate muscle injury such as exertional heat stroke (EHS)-induced rhabdomyolysis.^38^ EHS is a life-threatening disease characterized by high mortality and incidence of rhabdomyolysis (RM) that caused by the rapid (rhabdo) skeletal muscle (myo) breakdown (lysis), resulting in the subsequent release of intracellular muscle components into the systemic circulation.^115^^,^^116^ Specifically, in an EHS-induced RM murine model, the expression of ACSL4 increased following the onset of EHS, pharmacological inhibition of ACSL4*,* or blocking lipid peroxidation prevented EHS-induced ferroptosis and ameliorated skeletal muscle tissue injury and significantly improved the mortality of EHS mouse, suggesting that ACSL4 plays a critical role in regulating the activation of ferroptosis in skeletal muscle cells via lipid peroxidation.^38^ Thus, targeting ACSL4 may represent a novel therapeutic strategy to limit cell death and prevent RM after EHS. In heart failure, the expression of ACSL4 in cardiomyocytes was elevated, which was shown to trigger ferroptosis and aggravate the cardiac hypertrophy by activating pyroptotic signaling.^39^

LOXs also promote ferroptosis via lipid peroxidation, producing hydroperoxide. For example, the inactivation of ALOX12 and a missense mutation in *ALOX12* were shown to reduce the enzyme’s ability to oxidize PUFAs and diminish p53-mediated ROS-induced ferroptosis.^112^ Moreover, the photosensitizer triphenylamine-modified cyan-phenylenevinylene derivative (TPCI) activates ALOX12 through co-localization, promoting the production of large numbers of lipid ROS and triggering ferroptosis.^117^ Notably, TPCI-induced ferroptosis mediated by ALOX12 activation does not require ACSL4,^117^ suggesting that ALOX12 activation may increase sensitivity to ferroptosis in cells with low levels of ACSL4 expression. Following myocardial ischemia/reperfusion (I/R) injury, ALOX15 metabolites accumulate in ferroptotic cardiomyocytes, and ferroptosis is significantly reduced in cardiomyocyte-specific *Alox15* knockout mice.^118^ Furthermore, 15-hydroperoxyeicosatetraenoic acid (15-HpETE), an intermediate metabolite of ALOX15, has been identified as a trigger for ferroptosis in cardiomyocytes. It promotes the ubiquitin-dependent degradation of peroxisome proliferator-activated receptor γ coactivator 1-α (PGC1α), resulting in decreased mitochondrial biogenesis and altered mitochondrial morphology.^118^ In addition, the, ALOX15-specific inhibitor ML351 was shown to increase PGC1α expression in cardiomyocytes, inhibiting ferroptosis, protecting the damaged myocardium, and promoting the recovery of cardiac function.^118^ ALOX15 has also been shown to as a so-called “burning point” during the ischemic phase of myocardial I/R injury, with ALOX15-activated PUFA-phospholipid peroxidation increasing susceptibility to ferroptosis in I/R-induced myocardial injury.^119^

It is worth noting that the myocardium has several potential sources of endogenous ROS, including the mitochondrial ETC and the enzymes xanthine oxidoreductase and NADPH oxidase.^120^ Therefore, cardiac muscle is highly susceptible to oxidative damage. Among the various manifestations of oxidative stress in cardiac muscle, lipid peroxidation is particularly significant, contributing to both the development and severity of heart disease.^121^ Destruction of the cell membrane structure by lipid peroxidation is a driving factor for myocardial cell ferroptosis, and preventing lipid peroxidation protects the structural integrity of myocardial cell membrane and inhibits the process of ferroptosis.^122,123^

### Antioxidant defense mechanisms in the muscular system and ferroptosis

Free radicals, including ROS and reactive nitrogen species (RNS), play a crucial role in regulating cell survival and death. Antioxidants protect cells by either directly scavenging these free radicals or indirectly consuming compounds that are prone to forming free radicals, thereby preventing further harmful reactions. Both enzymatic and non-enzymatic antioxidants are essential in safeguarding cells against damage induced by peroxidation.

#### The oxidant-antioxidant system

Oxidative metabolism produces a variety of small molecular products that can significantly affect physiological processes and pathological pathways, with ROS and RNS being key chemical components. Reduced GSH is an antioxidant tripeptide composed of glutamic acid, cysteine, and glycine, with cysteine serving as the rate-limiting precursor for GSH synthesis. System Xc^‒^ imports extracellular cystine in exchange for intracellular glutamate,^124^ rapidly reducing cystine to form cysteine, which is then used in the synthesis of GSH.^125^ Regarded as a heterodimeric amino acid antiporter at the cell surface,^126,127^ system Xc^‒^ consists of two subunits: SLC7A11 (solute carrier family 7A member 11, also known as xCT) and SLC3A2 (also known as 4F2hc).^128,129^ The extracellular and intracellular concentrations of cystine and glutamate maintain the function of system Xc^‒^ to determine the redox state of cells. Mitochondria, being the main site of oxidation reactions, must maintain sufficient levels of GSH to perform biosynthesis functions. Since GSH is synthesized exclusively in the cytoplasm, the molecules responsible for its transport into mitochondria are particularly important. Chen et al. identified dicarboxylate carrier (SLC25A10) and oxoglutarate carrier (OGC, also known as SLC25A11) as participants in the mitochondrial transport of GSH.^130^ Subsequent studies have also found that in liver and retinal cells, SLC25A10 and SLC25A11 are involved in the maintenance of mitochondrial GSH, while the inhibition of these two transporters results in reduced mitochondrial GSH levels, ultimately leading to mitochondrial dysfunction.^131–133^ However, these conclusions contradict the results presented by Booty et al. ^134^, suggesting that the function of SLC25A10 and SLC25A11 as mitochondrial GSH transporters remains to be demonstrated. SLC25A22 has also been reported to be involved in cystine transport and GSH synthesis.^135,136^ Additionally, Wang et al. employed organellar proteomics and metabolomics to identify SLC25A39 may act as a regulator of GSH transport to mitochondria.^137^ This finding was further confirmed by Shi et al., who demonstrated that SLC25A37-mediated mitochondrial iron uptake and SLC25A39-mediated GSH homeostasis jointly sustain mitochondrial oxidative phosphorylation (OXPHOS).^138^ GPX4 converts reduced GSH into oxidized GSH and moderates lipid hydroperoxides (L-OOH) to lipid alcohols (L-OH) to minimize free radicals-related cellular damage.^13^ Thus, an increase in GPX4 expression diminishes the iron-dependent formation and accumulation of toxic lipid ROS, while inactivation of GPX4 accelerates the process of lipid ROS overload.^139^

NADPH serves as a crucial reducing agent, synthesized predominantly via the pentose phosphate pathway or through phosphorylating NADH. It plays a pivotal role in mitigating oxidative damage triggered by free radicals, as it can limit peroxidation-related damage.^140^ Many antioxidant enzymes, such as GPX4, FSP1, and NOXs, utilize the NADPH system to regulate electron transport,^141,142^ underscoring NADPH’s essential role in antioxidant processes. The myristoylation of FSP1 enhances its translocation from the mitochondria to the plasma membrane, where it functions as an NADH-dependent CoQ10 oxidoreductase, suppressing lipid peroxidation through CoQ10 reduction.^143^ Additionally, peroxidases, which are a Se-independent family of GSH peroxidases, also contribute to alleviating oxidative stress.

#### The oxidant-antioxidant system regulates ferroptosis in the muscular system

In addition to iron homeostasis disorder, ROS production induced by various stimuli is also one of crucial elements in the initiation of ferroptosis. Excess iron induces the production of a large number of free radicals through the Fenton reaction, which involves the interaction between hydrogen peroxide and a transition metal, usually iron (Fe^2+^), resulting in the production of highly reactive hydroxyl radicals (·OH). It is worth noting that there are many reasons for ROS accumulation, and the Fenton reaction activated by iron overload is one of them. Mitochondria are the main source of intracellular ROS, and superoxide anions are produced by oxidative phosphorylation in mitochondria, and superoxide dismutase (SOD) in mitochondria can also convert superoxides into other reactive oxygen species, including hydrogen peroxide.^144,145^ The large number of mitochondria in muscle cells and the high energy demand caused by muscle contraction led to a large number of mitochondrial ROS in muscle cells.^42,43^ In addition, overexpression of NOX can increase ROS levels and enhance ferroptosis sensitivity.^146^ ROS can be a byproduct of enzymatic reactions such as cytochrome P450 oxidoreductase, which is involved in drug metabolism, inducing lipid peroxidation and ferroptosis by producing superoxide free radicals.^113,147^

Both reduced cellular antioxidant capacity due to GSH deficiency and an inactivation of GPX4 have also been shown to promote ferroptosis.^91^ Furthermore, elevated levels of extracellular glutamate can restrict the uptake of cystine, thereby gradually promoting ferroptosis. Therefore, the activation of system Xc^−^ is essential in preventing ferroptosis. For instance, studies have demonstrated that overexpression of SLC7A11 in cardiomyocytes can increase cellular GSH levels and reduce FTH deficiency-mediated cardiac ferroptosis.^37^ Moreover, the accumulation of extracellular glutamate inhibits system Xc^−^, serving as a natural trigger to ferroptosis under physiological conditions.^35,91^

Like intracellular GSH,^148^ mitochondrial GSH may also contribute to inhibiting ferroptosis. For example, the ferroptosis inducer RSL3 was shown to induce mitochondrial fragmentation and lipid peroxidation in cardiomyocytes. LC-MS/MS analysis revealed a significant increase in mitochondrial levels of ferroptosis-promoting oxygenated phosphatidylethanolamine, while inhibiting oxidative phosphorylation of the electron transport chain drastically enhanced RSL3-induced cardiomyocyte ferroptosis.^149^ Furthermore, inhibition of SLC25A10 and SLC25A11 could reduce mitochondrial GSH content, increase mitochondrial ROS, and promote ferroptosis in cardiomyocytes.^149^

While GSH/GPX4 is widely considered the principal inhibitor of ferroptosis, recent studies have identified several other antioxidant systems that can inhibit ferroptosis independently of GPX4. For example, both FSP1/CoQ10 and GTP cyclohydrolase 1/tetrahydrobiopterin (GCH1/BH4) pathways have been shown to prevent ferroptosis independent of GPX4.^143,150–152^ Compared to control cells, cells deficient in FSP1 exhibit heightened sensitivity to ferroptosis-inducing agents such as the GPX4 inhibitor ML162 and the system Xc^‒^ inhibitor erastin. FSP1 functions as an NADH-dependent CoQ10 oxidoreductase, reducing CoQ10 to prevent lipid peroxidation and ferroptosis.^143^ Additionally, cells expressing GCH1 produce BH4/dihydrobiopterin (BH2), which facilitates lipid remodeling and selectively prevents the depletion of phospholipids containing two polyunsaturated fatty acid acyl tails, thereby inhibiting ferroptosis.^151,153^ Furthermore, other endogenous metabolites such as indole-3-pyruvate may inhibit ferroptosis by either neutralizing free radical intermediates required for lipid peroxidation or by regulating the expression of genes that control lipid peroxidation.^154^

### Molecular regulation of ferroptosis in the muscular system

**Fig. 3:**
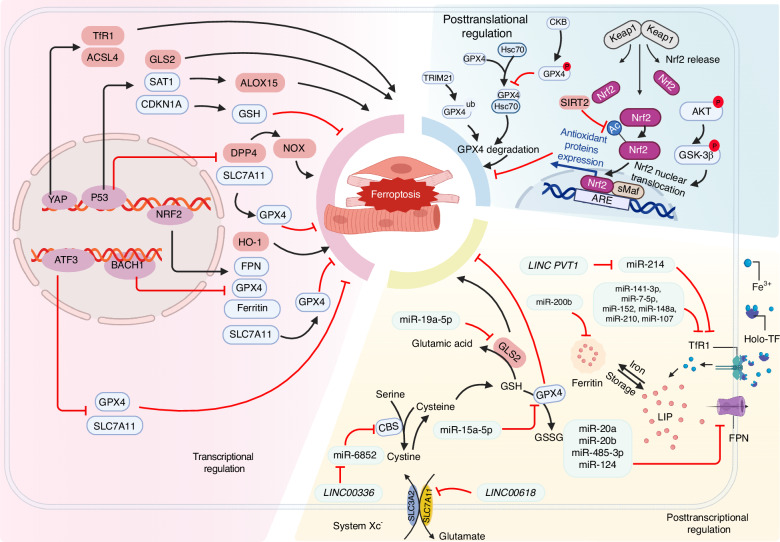
Summary of the signaling pathways that regulate ferroptosis in muscle cells. Several transcription factors such as p53, Nrf2, ATF3, and YAP regulate the transcription of ferroptosis-related genes. Noncoding RNAs are also involved in the posttranscriptional regulation of ferroptosis-related genes, affecting their expression. Acetylation and phosphorylation of Nrf2 regulate its nuclear translocation, thereby regulating the expression of downstream target genes such as *SLC7A11*, *GPX4*, and *FPN*. This figure was created with BioRender (https://biorender.com/). Abbreviations: ACSL4 acyl-CoA synthetase long-chain family member 4, ARE antioxidant response element, ATF3 activating transcription factor 3, BACH1 BTB domain and CNC homolog 1, CBS Cystathionine β-synthase, CDKN1A cyclin-dependent kinase inhibitor p21, DPP4 dipeptidyl peptidase 4, FPN ferroportin, GLS2 glutaminase 2, GPX4 glutathione peroxidase 4, GSH glutathione, HO-1 heme oxygenase 1, Keap1 Kelch-like ECH-associated protein 1, LIP labile iron pool, NOX NADPH oxidase, Nrf2 nuclear factor erythroid 2-related factor 2, SAT1 spermidine/spermine N1-acetyltransferase 1, Sirt2 Sirtuin-2, SLC7A11 solute carrier family 7 member 11, TF transferrin, TfR1 transferrin receptor protein 1

Ferroptosis is regulated by a network of organelles in which cellular mechanisms integrate multiple pro-ferroptosis and/or anti-ferroptosis signals at various levels in order to determine whether or not to initiate ferroptosis. Here, we outline the evidence to date supporting the role of various ferroptosis-related proteins in either triggering or preventing ferroptosis in the muscular system (Fig. 3), and we explore their potential relevance to muscle diseases and disorders.

#### Translational regulation of ferroptosis

Transcription factors such as p53 and Nrf2 play critical roles in the regulation of ferroptosis.^155^ Interestingly, p53 exhibits a dual role in this process. On one hand, p53 inhibits ferroptosis by suppressing the activity of the enzyme dipeptidyl peptidase 4 and inducing expression of the cyclin-dependent kinase inhibitor p21. On the other hand, p53 promotes ferroptosis by downregulating the expression of SLC7A11 and upregulating the expression of SAT1 (spermidine/spermine *N*^1^-acetyltransferase 1) and glutaminase 2. These opposing, context-dependent effects of p53 are likely determined by the intracellular oxidative stress state of cells.^156^ Nrf2 is a well-known transcription factor with antioxidant effects, and its downstream genes include *HMOX1* (which encodes HO-1), as well as genes involved in regulating GSH and iron;^157^ thus, Nrf2 is considered an important regulator of ferroptosis.^158,159^ For instance, Nrf2 can directly bind to the promoter sequence of *SLC7A11* and increase its expression.^160^ Nrf2 also affects—either directly or indirectly—the expression of TfR1, ferritin, and FPN in order to modulate iron storage, export, and catabolism. Upregulation of Nrf2 inhibits both erastin- and RSL3-induced ferroptosis in rat neonatal cardiomyocytes.^161,162^ In addition, Nrf2 maintains iron homeostasis by regulating the expression of *HERC2* and *VAMP8* (encoding E3 ubiquitin protein ligase 2 and vesicle-associated membrane protein 8, respectively).^163^ These results suggest that the Nrf2 signaling pathway plays an important role in ferroptosis and helps maintain iron homeostasis through the regulation of GSH homeostasis, lipid metabolism, and iron transport.

The transcription factor activating transcription factor 3 (ATF3) inhibits *SLC7A11* expression by binding to its promoter, thereby depleting intracellular GSH and promoting erastin-induced lipid peroxidation.^164^ Moreover, *Atf3* knockout significantly elevates the expression of SLC7A11 and GPX4.^165^ Yes-associated protein (YAP) is an important transcriptional co-activator in the Hippo signaling pathway to promote ferroptosis by upregulating several modulators of ferroptosis such as TfR1 and ACSL4.^166^ In H9c2 cells (a rat cardiomyocyte cell line), doxorubicin (DOX)-induced upregulation of YAP expression in cardiomyocytes is accompanied by an upregulation of ACSL4 and a downregulation of GPX4, thereby inducing ferroptosis. Conversely, inhibition of YAP activity, either by using an inhibitor or siRNA-mediated knockdown, attenuates both mitochondrial lipid peroxidation and ferroptosis.^167^ In EHS-induced rhabdomyolysis, ferroptosis in skeletal muscle cells is dependent on YAP-mediated upregulation of ACSL4.^38^ In addition, YAP can regulate ferroptosis by modulating the expression of FSP1,^168^ and an accumulation of endogenous glutamate increases the cell’s sensitivity to ferroptosis by inhibiting YAP.^169^ Although YAP does not directly regulate the transcription of *SLC7A11*, the YAP/TAZ complex maintains the protein stability, nuclear localization, and transcriptional activity of ATF4, thereby synergistically inducing the expression of SLC7A11.^170^ In vascular smooth muscle cells, YAP1 can promote the synthesis of glutamate and GSH by regulating the expression of glutaminase 1 (GLS1) and inhibit ferroptosis in smooth muscle cells.^171^

The heme-regulated transcription factor BTB and CNC homology 1 (BACH1) represses the expression of genes involved in iron and heme metabolism. BACH1 inhibits target genes critical for iron utilization and mobilization, including those encoding ferritin and FPN, as well as genes involved in the synthesis of GSH. This repression increases the susceptibility of cardiac muscle cells to ferroptosis.^172,173^ Importantly, *Bach1* knockout mice demonstrate greater resistance to myocardial infarction compared to wild-type counterparts.^173^ BACH1 promotes ferroptosis by disrupting the balance between the induction of protective genes and the accumulation of iron-mediated damage at the transcriptional level. This suggests that BACH1 sets a critical threshold for ferroptosis induction and may serve as a therapeutic target in treating ferroptosis-related diseases and conditions, including myocardial infarction.

#### Posttranscriptional regulation of ferroptosis

Posttranscriptional regulation encompasses the splicing and processing of pre-mRNA, the processing and localization of mRNA from the nucleus to the cytoplasm, and the stabilization and degradation of mRNA. MicroRNAs (miRNAs) can regulate ferroptosis by modulating the mRNA levels of genes involved in iron metabolism, thereby maintaining iron homeostasis. For example, increased expression of the miRNAs miR-20a and miR-124 inhibits the expression of FPN, leading to the accumulation of intracellular iron.^174–177^ Overexpression of miR-200b suppresses ferritin expression, consequently reducing iron storage.^178^ In addition, upregulation of myocardial miR-15a-5p, which targets *GPX4* mRNA, has been observed in a mouse model of acute myocardial infarction.^179^ This overexpression enhances ferroptosis, in turn exacerbating hypoxic damage in cardiomyocytes. Conversely, reducing miR-15a-5p levels increases GPX4 expression, thus attenuating ferroptosis and offering protection against myocardial injury.^179^ Additionally, miR-190a-5p was shown to reduce ferroptosis in H9c2 cells by directly targeting *GLS2* (encoding glutaminase 2), reducing the accumulation of ROS, MDA, and Fe^2+^. Inhibition of this miRNA leads to an upregulation of GLS2, with increased susceptibility to ferroptosis.^180^

Previous studies have demonstrated that long noncoding RNAs (lncRNAs) are involved in various biochemical pathways related to cell death, including ferroptosis. For instance, the lncRNA *LINC00618* was shown to accelerate iron toxicity by elevating levels of lipid ROS and iron while reducing SLC7A11 expression.^181^ In addition, lncRNAs may regulate ferroptosis by acting as a competing endogenous RNA; for example, the lncRNA *PVT1* regulates ferroptosis by binding to the miRNA miR-214, thereby interfering with its inhibitory effects on *TfR1* mRNA.^182^ Interestingly, the lncRNA *LINC00336* serves as an endogenous “sponge” of miR-6852 to regulate the expression of *CBS*, which encodes cystathionine-β-synthase, a surrogate marker of ferroptosis.^183^ Similarly, the lncRNA *RP11-89* sponges miR-129-5p, upregulating the expression of *PROM2* (encoding the membrane glycoprotein prominin 2) to reduce ferroptosis through the formation of multivesicular bodies and increase iron export.^184^ Moreover, the cardiac‐related lncRNA *ZFAS1* (zinc finger antisense 1) has recently been associated with acute myocardial infarction, where inhibiting *ZFAS1* with shRNA reduces apoptosis and ferroptosis in cardiomyocytes, thereby mitigating diabetes-induced myocardial damage.^185^ These findings indicate that ferroptosis-related lncRNAs may serve as suitable biomarkers and/or therapeutic targets for maintaining iron homeostasis.

#### Epigenetic regulation of ferroptosis

Epigenetic regulation mainly involves DNA methylation and posttranslational modification of histones. DNA methylation is the most widely studied epigenetic modification and plays an important role in the regulation of gene expression. The Ten-eleven translocation (TET) family are iron (Fe^2+^)- and α-ketoglutaric acid (α-KG)-dependent dioxygenases, with TET2 acting as DNA demethylase that enhances the transcription activity of promoter CpG islands and promotes gene expression. TET2-mediated DNA demethylation can directly or indirectly elevate the expression of FPN and Erythroferrone by combating oxidative stress through demethylation.^186^ In contrast, knockdown or reduction of *Tet2* expression could affect the methylation of CpG sites in genes related to iron metabolism and heme biosynthesis, such as Fech, Abcb7 and Sf3b1, leading to suppressed expression and subsequent iron accumulation.^187,188^ In addition, the aberrant expressions of TfR2 and hepcidin has been linked to abnormal methylation of their promoter regions. Treatment with demethylating drugs could reverse the hypermethylation of the hepcidin promoter, thereby upregulating the expression of hepcidin and regulating the efflux of iron.^189,190^ These findings suggest DNA methylation may control the expression of hepcidin and other iron-sensing genes, impacting iron homeostasis at the epigenetic level.

N6-methyladenosine (m6A) methylation is the most prevalent type of RNA modification. The fat mass and obesity-associated protein (FTO) inhibits DOX-induced ferroptosis in cardiomyocytes through p21/Nrf2 activation by mediating m6A demethylation of p21/Nrf2.^191^ FTO also regulates the m6A modification of BACH1 and is involved in septic cardiomyopathy through inhibiting ferroptosis.^192^ Oxygen glucose deprivation/recovery (OGD/R) could induce the upregulation of methyltransferase-like 3 (METTL3) in cardiomyocytes, where METTL3 binds to SLC7A11 to promote the m6A methylation of SLC7A11.^193^ In addition, YTH domain-containing family protein 2 (YTHDF2) acts as a reader recognizing the methylation of SLC7A11, and the silencing of METTL3 inhibits OGD/R-induced ferroptosis by preventing the YTHDF2-related m6A methylation of SLC7A11.^193^ In smooth muscle cells, the lncRNA *NORAD* stabilizes *GPX4* mRNA and elevates GPX4 levels, leading to inhibition of angiotensin II (Ang II)-induced ferroptosis of vascular smooth muscle cells (VSMCs) and aortic dissection. METTL3 also enhances m6A methylation of *NORAD* in a YTHDF2-dependent manner.^194,195^

Histone acetylation is a reversible histone modification that plays an important role in regulating eukaryotic gene expression and chromatin structure and function. This process is dynamically controlled by histone acetyltransferase and histone deacetylase (HDAC). In the brain, iron accumulation has been shown to reduce the level of H3K9 acetylation in the hippocampus, leading to memory impairment and neurodegenerative diseases.^196^ Meanwhile, HDAC inhibitors can enhance the binding of the transcription factor Sp1 to its promoter region nuclear factor, thereby inducing the expression of ferritin heavy chain and affecting iron storage.^197^ SIRT2, a cytoplasmic deacetylase, promotes the deacetylation of Nrf2, resulting in decreased FPN expression and preventing the efflux of intracellular iron.^198^ Hepcidin histone deacetylation has been found to downregulate the expression of hepcidin and promote iron absorption.^199^ Overexpression of mothers against decapentaplegic homolog 4 (SMAD4) or HDAC1 inhibitor to increase histone H3K9 acetylation at the hepcidin promoter upregulates the expression of hepcidin, effectively alleviating the symptoms of iron accumulation, showing potential for treating diseases associated with iron overload.^200,201^ These findings underscore the significant role that histone acetylation-related proteins play in regulating iron homeostasis.

In addition, histone H3K4me1 and H3K4me2 levels were significantly reduced in the aorta of mice with aortic dissection. SP2509, a specific inhibitor of lysine-specific demethylase 1 (LSD1) in VSMCs, results in a dose-dependent increase in H3K4me2 levels.^202^ SP2509 has a protective effect on the ferroptosis of VSMCs, which can be demonstrated by increasing cell viability and reducing cellular lipid peroxidation. More importantly, SP2509 inhibits the expression of TfR and ferritin, leading to reduction of intracellular iron levels, thereby effectively blocking ferroptosis of VSMCs.^202^ The histone methyltransferase inhibitor BRD4770 also shows a protective effect against cysteine deprivation and RSL3-induced ferroptosis in smooth muscle cells.^203^

#### Posttranslational modifications of ferroptosis

Posttranslational protein modifications such as phosphorylation, polyubiquitination, and acetylation play vital roles in regulating protein activity, stability, and/or folding. These modifications are essential in controlling ferroptosis by modulating the activation and inactivation of a variety of ferroptosis-related signaling molecules, such as GPX4 and FTH. For instance, creatine kinase B phosphorylates GPX4 at S104, preventing the chaperone protein HSC70 from binding to GPX4, reducing its degradation, and inhibiting ferroptosis.^204^ In mouse hearts or neonatal rat cardiomyocytes treated with DOX, downregulation of AMP-activated protein kinase (AMPK) phosphorylation was observed, accompanied by upregulation of ferroptosis-related protein genes. Phosphorylation of AMPK induces phosphorylation of aminocyclopropane-1-carboxylic acid (ACC), inhibiting lipid peroxidation and ferroptosis of cardiomyocytes.^205,206^ HIP-55 is a novel adaptor protein to suppress ferroptosis in cardiomyocytes, and deletion of HIP-55 gene increases susceptibility to ferroptosis. Protein kinase B (AKT/PKB) phosphorylates HIP-55 at S269/T291, which negatively regulates the mitogen-activated protein kinase kinase kinase kinase 1 (MAP4K1) pathway to counteract ferroptosis of cardiomyocytes.^207^ Additionally, phosphorylation of AKT and glycogen synthase kinase 3β (GSK-3β) activates the nuclear translocation of Nrf2, leading to increased expression of downstream targets such as GPX4, SLC7A11 and TfR1. This process reduces iron accumulation and occurrence of ferroptosis in mice.^208^

The HECT domain-containing ubiquitin E3 ligase1 (HUWE1) specifically targets TfR1 for ubiquitination and proteasome degradation. Knockout of *HUWE1* leads to increased intracellular TfR1 levels, promoting ferroptosis and exacerbating liver injury caused by I/R and CCL_4_.^209^ In addition, inhibition of HUWE1 also significantly enhances the cellular sensitivity of primary hepatocytes to ferroptosis.^209^ The tripartite motif containing 21 (TRIM21) ubiquitinates GPX4, enhancing ferroptosis and aggravating I/R-induced acute kidney injury.^210^ Ubiquitination of histone 2A (H2Aub) binds at SLC7A11 promoter is generally associated with transcriptional repression. Ubiquitin carboxyl-terminal hydrolase BAP1 is an H2A deubiquitinase that reduces SLC7A11 expression by decreasing H2A ubiquitination, thereby inhibiting cystine uptake and leading to increased lipid peroxidation and ferroptosis.^211^ The E3 ubiquitin ligase FBXW7 targets ZFP36 (zinc finger protein 36 homolog) for ubiquitination and degradation, which promotes autophagy-related 16 like 1 (ATG16L1) expression and autophagy to target the degradation of ferritin and the release of iron, ultimately leading to ferroptosis.^212^ Interestingly, the natural polyphenol resveratrol was shown to inhibit ferroptosis and reduce myocardial I/R injury by regulating ubiquitin-specific peptidase 19 (USP19)/Beclin 1-mediated autophagy.^213^ Some mitochondrial proteins, including NFS1, are involved in iron-sulfur cluster biogenesis and negatively modulate ferroptosis, possibly by reducing the available redox-active iron content, while NFS1 deficiency increases susceptibility to ferroptosis in cardiomyocytes.^11^ DOX downregulates GPX4 and NFS1 expression, inducing ferroptosis in cardiomyocytes by promoting mitochondrial membrane protein optic atrophy 3 (OPA3) ubiquitination. However, exogenous H_2_S antagonizes OPA3 ubiquitination by promoting OPA3 S-sulfation and upregulating NFS1, thereby inhibiting ferroptosis and preventing DOX-induced cardiotoxicity.^214^

SUMOylation—a process by which SUMO (small ubiquitin-related modifier) proteins are covalently attached to specific lysine residues on target proteins—may affect ferroptosis in H9c2 cells by modulating key ferroptosis regulators such as hypoxia-inducing factor (HIF-1α), ACSL4, and GPX4. In cardiomyocytes, the de-SUMOylation of ACSL4 has been shown to play a critical role in erastin-induced ferroptosis.^215^ Acetylation is an important post-translational modification involved in many cell signaling pathways and diseases, and plays a key role in ferroptosis.^216^ For example, acetylation of p53 can promote ferroptosis in cardiomyocytes, while SIRT3 can alleviate ferroptosis by inhibiting p53 acetylation.^217^ ALOX12 is a key protein to initiate membrane phospholipid oxidation. The ferroptosis inducer RSL3 promotes both expression and acetylation of ALOX12, thereby inducing ferroptosis in myoblasts and skeletal muscle cells. Reducing ALOX12 acetylation decreases membrane lipid peroxidation and prevents RSL3-induced ferroptosis in skeletal muscle cells.^218^ Moreover, the acetylation status of transcription factor Sp1 is related to the occurrence of ferroptosis. Acetylated Sp1 cooperates with transcription factor AP-2 gamma (TFAP2c) to initiate the transcription response to ferroptosis-related proteins, particularly GPX4. Notably, selective inhibitors of HDAC have shown beneficial effects in preventing ferroptosis.^219,220^

### Crosstalk between ferroptosis and other forms of cell death in the muscular system

Although ferroptosis is both morphologically and biochemically distinct from other forms of cell death,^221^ these various forms of cell death can occur simultaneously, are not necessarily independent, and may overlap or engage in crosstalk, further complicating their interactions.^22,222^ For instance, cardiomyopathy induced by chemotherapy agents such as DOX often restricts their clinical application and is associated with a poor prognosis. While sirtuins (signaling proteins involved in metabolic regulation) have been reported to be involved in autophagy, apoptosis, and pyroptosis in DOX-induced cardiomyotoxicity, they have also been linked to ferroptosis resulting from iron overload.^198,223^

#### Crosstalk between ferroptosis and apoptosis in the muscular system

DOX-induced cardiotoxicity is associated with both ferroptosis and apoptosis in cardiomyocytes. The combination of Ferrostatin-1 (Fer-1), a ferroptosis inhibitor, and zVAD, an apoptosis inhibitor, completely prevents DOX-induced cardiomyocyte death.^224^ Moreover, the ferroptosis inhibitor liproxstatin-1 was shown to prevent apoptosis in cardiomyocytes subjected to a high-fat diet.^225^ The anesthetic compound propofol also activates the Nrf2/GPX4 signaling pathway and simultaneously attenuates DOX-induced ferroptosis and apoptosis.^226^ Arsenic trioxide (ATO) induces both ferroptosis and apoptosis in cardiomyocytes, with Fer-1 demonstrated to inhibit ATO-induced apoptosis.^227^

#### Crosstalk between ferroptosis and pyroptosis in the muscular system

Pyroptosis is triggered by pro-inflammatory signals and is mediated by gasdermin proteins and cysteine-aspartate-specific caspases. Mixed lineage kinase 3 (MLK3), a member of the mitogen-activated protein kinase kinase kinase (MAP3K) family, is associated with myocardial diseases such as congestive heart failure. Activation of the MLK3 signaling pathway in cardiomyocytes was shown to induce both pyroptosis and ferroptosis, promoting myocardial fibrosis.^228^ Inflammasome NLR family pyrin domain containing 3 (NLRP3)-mediated pyroptosis has been reported to induce ferroptosis in diabetic cardiomyopathy;^229^ specifically, in diabetic mice with myocardial injury, upregulation of the macrophage migration inhibitory factor (MIF)/CD74 axis in myocytes activates NLRP3, leading to pyroptosis, significant downregulation of GPX4, and depletion of GSH, ultimately inducing ferroptosis.^229^ In addition, the pyroptosis inhibitors MCC950 and necrosulfonamide were shown to mitigate the alterations in GPX4, GSH, and lipid peroxidation observed in myocardial cells in diabetic mice. Inhibiting NLRP3 and pyroptosis reversed high-sugar/high-fat‒induced cardiomyocyte dysfunction, similar to the effects of liproxstatin-1 and the mitochondrial antioxidant MitoQ.^229^ This suggests that ferroptosis in cardiomyocytes in diabetic cardiomyopathy is regulated by NLRP3-dependent pyroptosis. Recent study has also found that in cardiomyocytes, ACSL4-dependent ferroptosis drives activation of pyroptosis, causing heart failure.^39^

#### Crosstalk between ferroptosis and autophagy in the muscular system

Autophagy is a process that involves transporting cytoplasmic cargo to lysosomes for degradation and recycling. Ferritinophagy degrades ferritin in lysosomes and regulates iron metabolism to maintain intracellular iron balance, however, excess ferritinophagy leads to the increase and deposition of free iron. The relationship between NCOA4-mediated ferritinophagy and ferroptosis has been extensively studied.^58,230^ In cardiomyocytes, PM2.5 can not only promote the accumulation of unstable iron in the cells and mitochondria caused by ferritinophagy, leading to lipid peroxidation and mitochondrial dysfunction, but also activate mitochondrial autophagy, enhancing the sensitivity of cardiomyocytes to ferroptosis. It is noteworthy that abnormal iron metabolism mediates the activation of ferritinophagy and mitochondrial autophagy in a chronological order in PM2.5-induced ferroptosis, suggesting that the crosstalk among ferritinophagy, mitochondrial autophagy and ferroptosis plays an important role in PM2.5-induced myocardial hypertrophy.^231^ In smooth muscle cells (SMCs), hypoxiainduced an increase in lncRNA *MIR210HG* level and promoted the transition of SMCs from contractile phenotype to synthetic phenotype by activating the autophagy-dependent ferroptosis pathway.^232^

## Regulation of systemic metabolism by the muscular system

Recent evidence suggests that muscles, beyond their traditional roles in maintaining posture and movement, act as endocrine organs that affect systemic metabolism by releasing muscle factors.^233,234^ Muscle fibers secrete cytokines and other peptides such as “myokines” from skeletal muscle and “cardiokines” from cardiac muscle, which not only respond to muscle contraction but also affect the metabolism of other tissues and organs, thus playing a crucial role in regulating energy homeostasis throughout the body.^235,236^ Over the past few decades, studies have shown that skeletal muscle secretes hundreds of myokines, which exhibit autocrine, paracrine, and/or endocrine activity.^2,237^ For example, interleukin 6 (IL-6) was the first myokine to be identified, and a growing body of evidence highlights its critical roles in metabolizing fatty acids and glucose in muscles and other organs following exercise.^233,238^ Moreover, muscle-released IL-6 has been shown to regulate energy metabolism and immune function, with exercise enhancing its release.^239,240^ As the predominant myokine, lactate plays an important role in promoting metabolism and maintaining health.^241^ In addition, lactate regulates the uptake and oxidation of fatty acids, stimulates the release of brain-derived neurotrophic factor in the brain to support neurogenesis and cognitive function,^242^ and affects hunger and appetite through the regulation of ghrelin-mediated signaling.^243^ Cardiokines such as atrial natriuretic peptide and ventricular natriuretic peptide also contribute to systemic metabolism by promoting the “browning” of white adipose tissue.^244^

Studies have demonstrated that muscle-secreted factors participate in many physiological and pathological processes by regulating ferroptosis. Irisin is a polypeptide hormone secreted by muscles that mediates several metabolic processes throughout the body as an endocrine factor, with exercise increasing the circulating concentration of irisin in both humans and rodents.^245^ Interestingly, serum levels of irisin are decreased in patients with sepsis. In septic mice, treatment with exogenous irisin reduced ROS production, reversed abnormal mitochondrial morphology, and inhibited liver ferroptosis.^246^ Moreover, the protective effects of irisin against ferroptosis in hepatocytes were diminished when the ferroptosis regulator GPX4 was inhibited.^246^ Irisin has also been shown to alleviate sepsis-related complications by inhibiting ferroptosis in hippocampal neurons and renal epithelial cells, primarily through the activation of the Nrf2/GPX4 signaling axis and the SIRT1/Nrf2 pathway.^247^ Furthermore, irisin exerts significant protection against I/R-induced lung injury and myocardial injury, with the underlying mechanism involving activation of the Nrf2/HO-1 axis, which helps reverse mitochondrial damage and reduce ferroptosis in hypoxic cells.^248,249^ Irisin can increase the expression of SLC7A11 and GPX4 through SIRT1-mediated p53 deacetylation, inhibit ferroptosis and improve diabetic cardiomyopathy.^250^ Irisin elevated during exercise also plays a vital role in maintaining bone health, not only inhibiting bone resorption, but also promoting bone growth.^251,252^ For example, irisin treatment significantly reduces lipid peroxidation and iron overload, and improves bone loss caused by diabetes by inhibiting ferroptosis.^253^ Irisin directly binds to the caveolin-1 (Cav1) of osteoblast and promotes the transcription of HO-1 and FPN by increasing AMPK/Nrf2 pathway, thereby inhibiting ferroptosis in osteoblasts and promoting osteoblast proliferation.^254^ In addition, exosomes secreted by myocytes are involved in the transport of irisin, and irisin enters osteoblasts through caveolae-mediated endocytosis.^254^ These results provide new insights into the mechanisms by which exercise improves osteoporosis.

Growth differentiation factor 8 (GDF-8), also known as myostatin (MSTN), is highly expressed in skeletal muscle tissue and acts as negative regulator of muscle development.^255^ Functional analyses have shown a reduction in ferroptosis pathways in MSTN-edited sheep.^256^ In a mouse model of chronic obstructive pulmonary disease (COPD), muscle tissue exhibited an enriched ferroptosis pathway, accompanied by increased expression of MSTN. MSTN upregulates the expression of HIF-2α, leading to elevated levels of Fe^2+^, lipid ROS, and 4-hydroxynonaldehyde (4-HNE), as well as reduced levels of GPX4 and GSH, while suppression of MSTN by binding to its receptor or inhibiting/knocking down HIF-2α was shown to decrease ferroptosis. These results suggest that MSTN may contribute to muscle dysfunction in COPD mice by impairing metabolic capacity and promoting ferroptosis. GDF11 and MSTN are closely related members of the transforming growth factor beta (TGF-β) superfamily and are often believed to have similar or overlapping roles.^257^ However, recent studies indicate that GDF11 and MSTN may have distinct roles in regulation of ferroptosis. Specifically, GDF11 may reduce ferroptosis in neurons by downregulating NCOA4 and LC3II, upregulating FTH1 and p62, thereby inhibiting ferritinophagy.^258^

Fibroblast Growth Factor 21 (FGF21) is a peptide hormone synthesized by several organs such as skeletal muscle and myocardium with pleiotropic effects on glucose and lipid homeostasis to maintain energy balance.^259,260^ Iron overload promotes ferroptosis in liver cells by inducing HO-1 expression, leading to liver fibrosis. The loss of FGF21 exacerbates ferroptosis caused by iron overload, while overexpression of FGF21 inhibits ferroptosis in hepatocytes primarily by promoting ubiquitination and degradation of HO-1 and activating Nrf2.^261^ Grape seed proanthocyanidins have also been found to reduce liver cell ferroptosis by enhancing the interaction between Nrf2 and FGF21.^262^ In the central nervous system, FGF21 can downregulate HO-1, increase GPX4 expression, and reduce iron deposition to inhibit ferroptosis, thereby improving spinal cord injury and promoting neurological recovery.^263^ In contrast, the FGFR1 (FGF21 receptor) inhibitor PD173074 partially reverses the therapeutic effect induced by FGF21.^264^ In myocardial tissue, FGF21 binds to ferritin to reduce its excessive degradation through proteasome and lysosome-autophagy pathways, thus inhibiting ferroptosis and diabetic cardiomyopathy, which may also be a positive feedback loop for FGF21 to regulate myocardial function.^265^

Apelin is a polypeptide hormone produced by skeletal muscle cells and cardiomyocytes that regulates the physiological function of cells by binding to its receptor (APJ).^266^ The Apelin/APJ signaling pathway plays a crucial role in several physiological and pathological processes. For example, it is involved in the regulation of the cardiovascular system, including aspects of myocardial contractility, myocardial metabolism, vasomotor and blood pressure.^267,268^ In addition, the Apelin/APJ signaling pathway also plays an important role in the regulation of energy metabolism, exercise endurance, inflammatory response and immune activity.^269–273^ While Apelin itself has not been reported to regulate ferroptosis, elabela, another endogenous ligand of APJ, has been found to inhibit ferroptosis. In hypertensive mice induced by Ang II, the level of elabela decreased, whereas administration of elabela significantly mitigated Ang II-induced iron upregulation and lipid peroxidation. This protective effect was achieved by inhibiting cardiac IL-6/signal transducer and activator of transcription 3 (STAT3) signaling and activating xCT/GPX4 signaling, which prevented pathological myocardial remodeling and hypertension.^274^ Activation of the elabela-APJ axis also mitigated cerebral I/R injury by reducing iron deposition, alleviating lipid peroxidation and inhibiting ferroptosis in neurons.^275^ Other muscle-secreted factors such as β-Aminoisobutyric Acid (BAIBA) and secreted protein acidic and rich in cysteine (SPARC) have also been reported to ameliorate lung I/R injury or osteoarthritis by activating antioxidant systems and reducing lipid peroxidation, thereby inhibiting ferroptosis.^276,277^

## The role of ferroptosis in muscle diseases and disorders

**Fig. 4:**
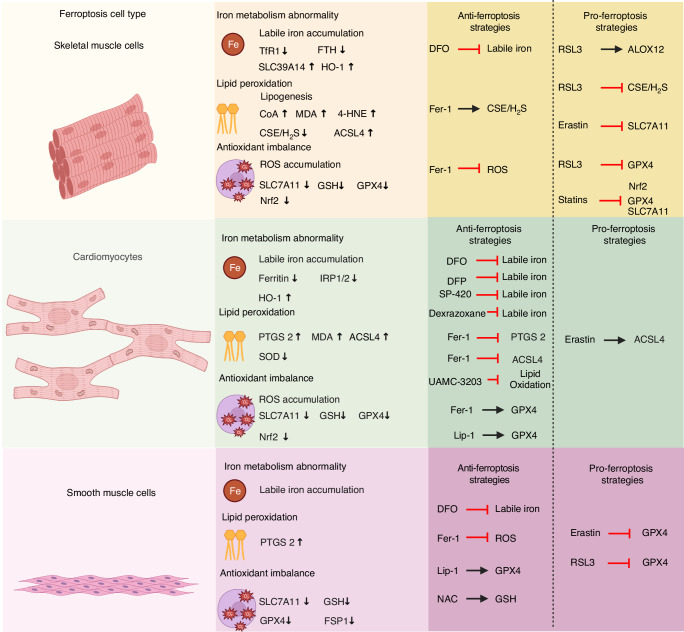
Strategies for targeting ferroptosis in treating muscle diseases and disorders. Dysregulated iron metabolism, lipid peroxidation, oxidation, and antioxidant imbalances have all been implicated in the development of a variety of diseases and disorders affecting muscles. These muscle diseases and disorders can be treated using either inhibitors (anti-ferroptosis strategies) or inducers (pro-ferroptosis strategies). This figure was created with BioRender (https://biorender.com/). Abbreviations: 4-HNE 4-hydroxynonaldehyde, ACSL4 acyl-CoA synthetase long-chain family member 4, DFO deferoxamine, DFP Deferiprone, Fer-1 ferrostatin-1, FSP1 ferroptosis suppressor protein 1, FTH ferritin heavy chain, GPX4 glutathione peroxidase 4, GSH glutathione, HO-1 heme oxygenase-1,IRP1/2 iron-regulated protein 1/2, Lip-1 liproxstatin-1, MDA malondialdehyde, NAC N-acetylcysteine, Nrf2 nuclear factor erythroid 2-related factor 2, PTGS2 prostaglandin-endoperoxide synthase 2, ROS reactive oxygen species, SLC7A11 solute carrier family 7 member 11, SLC39A14 metal cation symporter ZIP14, SOD superoxide dismutase, TfR1 transferrin receptor protein 1

Muscle diseases and disorders are a general term for abnormal pathological changes involving the structure and function of different types of muscle tissues, including skeletal muscle, myocardium, and smooth muscle. Muscle diseases and disorders encompass a wide range of conditions that can be either hereditary or acquired. Hereditary conditions, such as age-related sarcopenia, are caused by direct alterations within the muscle tissue itself.^278^ On the other hand, acquired conditions may arise from chronic diseases, metabolic disorders, or the effects of certain medications, including cancer-related muscular atrophy and chemotherapy-induced cardiomyopathy.^279,280^ In addition to severely affecting the patient’s quality of life and physical abilities, many muscle diseases and disorders such as sarcopenia, myocardial ischemia, and smooth muscle injury can increase the risk of acute or chronic conditions, including fractures, acute kidney injury, and diabetes.^281–283^ Therefore, understanding the pathogenesis of muscle diseases and disorders is urgently needed in order to identify new therapeutic targets. In this respect, exploring the role of ferroptosis in the progression of various muscle diseases and disorders may provide valuable insights for developing ferroptosis-targeted prevention and/or treatment strategies (Fig. 4 and Table 1).

### Ferroptosis in skeletal muscle diseases and disorders

#### Ferroptosis in age-related sarcopenia

Sarcopenia is a progressive, systemic disease marked by the loss of skeletal muscle mass and function, which significantly increases the risk of adverse outcomes, including falling, functional decline, and even death.^284^ Several physiological factors contribute to the onset and progression of sarcopenia,^285^ including physical inactivity,^286^ hormonal changes,^287^ malnutrition, inflammation, and oxidative stress.^288^ Emerging research has implicated ferroptosis contributes to in the disruption of skeletal muscle homeostasis, and the development of sarcopenia has been associated with an accumulation of iron within skeletal muscle tissues.^289,290^ Alterations in the expression of genes and/or proteins involved in iron regulation and metabolism have also been observed, with iron deposition leading to ferroptosis in both skeletal muscle cells and satellite cells, thereby contributing to the pathogenesis of sarcopenia.^23^

Interestingly, iron overload has been detected in the atrophic muscles of both patients with sarcopenia and animal models of the condition. In aged rats, reduced skeletal muscle mass is accompanied by increased iron accumulation,^291,292^ downregulated TfR1, and elevated ferritin expression.^289^ One of the mechanisms underlying skeletal muscle atrophy is increased proteasomal degradation.^293^ Iron loading has been shown to diminish the phosphorylation of AKT and forkhead box O3a (FOXO3a), leading to a loss of skeletal muscle mass. Importantly, reactivating the AKT-FOXO3a pathway by mitigating oxidative stress reverses skeletal muscle atrophy,^293^ highlighting the potential role of ferroptosis in this process.

A recent study found that iron accumulation in muscles induces ferroptosis by upregulating p53 and downregulating SLC7A11,^73^ leading to an increase in muscle cell death through the accumulation of lipid peroxidation products, which accelerates the progression of sarcopenia. These findings support the notion that iron accumulation and iron-induced lipid ROS drive ferroptosis, consequently altering skeletal muscle homeostasis and causing sarcopenia. In addition, these results suggest that inhibiting ferroptosis could potentially become part of the routine treatment for sarcopenia.

In addition to promoting skeletal muscle atrophy, ferroptosis also impedes the regeneration of skeletal muscle. Satellite cells, which are muscle stem cells residing beneath the basal lamina of myofibers, demonstrate the capacity for self-renewal and differentiation. When skeletal muscle is damaged, for example, due to trauma, these satellite cells proliferate and differentiate to repair the injured myotubes.^294^ The depletion of satellite cells in adult mice leads to significant muscle loss and a reduced capacity for tissue generation.^295^ Importantly, the progressive decline in both the number and function of satellite cells limits the regenerative potential of injured muscle. Recently, Ding et al. investigated the biological function of TfR1 in satellite cells and observed that the loss of *Tfr1* was associated with iron overload and mitochondrial dysfunction in aging mice. This was accompanied by elevated levels of SLC39A14, leading to an increased accumulation of unstable iron, which ultimately triggered ferroptosis. The ferroptosis inhibitor Fer-1 can inhibit ferroptosis in satellite cells, promoting muscle regeneration and enhancing the exercise ability of aging mice.^23^ However, Fer-1 treatment failed to reverse the ferroptosis induced by deletion of *Tfr1* in skeletal muscle.^23^ This may be attributed to the fact that *Tfr1* knockout in satellite cells is an irreversible process, leading to serious defects in the proliferation and differentiation of satellite cells, which cannot be compensated for by exogenous administration of Fer-1. Therefore, future studies are warranted to determine whether targeting SLC39A14 to rescue iron overload-induced skeletal muscle damage could offer therapeutic benefits.

#### Ferroptosis triggers amyotrophic lateral sclerosis

Redox metals like iron are involved in a wide range of metabolic processes in the brain, including neurotransmitter production and cell-cell communication.^296^ The oxidation of dopamine induced by iron modifies GPX4, rendering it susceptible to degradation via the ubiquitin-proteasome pathway. This degradation of GPX4, coupled with the preferential accumulation of phospholipid peroxide, results in damage to dopaminergic neurons, ultimately causing progressive motor dysfunction.^297^ ALS is a neurodegenerative disease characterized by the progressive loss and degeneration of motor neurons. Emerging evidence suggests that ferroptosis may contribute to this neurodegeneration. For example, single-nucleus RNA sequencing and bulk RNA sequencing data have identified ferroptosis and iron metabolism-related genes (FIRGs), including *ACSL4* and *CHMP5* (which encodes the chromatin-modifying protein-charged multivesicular body protein 5), are involved in the pathogenesis of ALS primarily by affecting calcium signaling pathways, synaptic pathways, and a variety of immune pathways.^298^ CHMP5 has also been shown to accumulate in the plasma membrane during ferroptosis, reducing lipid peroxidation and participating in the regulation of ferroptosis. Inhibition of CHMP5, however, was shown to increase ferroptosis.^299^ Previous studies have also found that elevated levels of CHMP5 in the blood correlate with reduced life expectancy in ALS patients, suggesting that CHMP5 could serve as a possible prognostic biomarker for ALS.^300^ These findings underscore a correlation between FIRGs and ALS, offering new avenues for research into the mechanisms underlying the loss of motor neurons in this disease.

Clinical studies have revealed a significant reduction in GPX4 expression in the spinal cord of patients with sporadic and familial ALS. This decrease in GPX4 compromises the anti-ferroptosis defense system, as evidenced by the inactivation of the Nrf2 signaling pathway and the dysregulation of ferritin in ALS patients and mouse models.^301,302^ Notably, overexpression of GPX4 in spinal cord motor neurons has been shown to significantly reduce muscle atrophy, enhance motor function, and extend survival, likely due to decreased lipid peroxidation and increased spinal motor neuron activity.^301,302^ On the other hand, GPX4 inhibition leads to irreversible lipid peroxidation and facilitates ferroptosis, and these effects can be reduced by overexpression of FSP1. Moreover, the paralysis and ferroptotic cell death of spinal motor neurons induced by *Gpx4* knockout were alleviated by inhibiting ferroptosis.^301,303^ Similarly, treatment with the ferroptosis inhibitor liproxstatin-1 reduced both paralysis and spinal motor neuron death in neuron-specific *Gpx4* inducible knockout (GPX4-NIKO) mice, and overexpression of the mitochondrial antioxidant defense enzyme peroxiredoxin 3 also ameliorated the symptoms in these mice.^304^ Together, these findings reinforce the idea that ferroptosis mediates the death of motor neurons in ALS, and strategies aimed at reducing ferroptosis-such as enhancing GPX4 activity or expression may represent promising therapeutic approaches for ALS.

#### Ferroptosis aggravates Duchenne muscular dystrophy

Duchenne muscular dystrophy (DMD) is an X-linked recessive disorder caused by mutations in dystrophin protein encoded by the *DMD* gene.^305^ Muscle cells lacking dystrophin are more sensitive to injury, resulting in abnormal production of ROS, a factor strongly associated with the severity of DMD and a common therapeutic target in muscular dystrophy. The accumulation of ROS, which drives lipid peroxidation, is considered the principal mechanism underlying sarcolysis in muscular dystrophy. Iron, a crucial modulator of oxidative stress, also contributes to dystrophic pathology.^306^ In a mouse model of DMD, the production of iron-dependent hydroxyl radicals has been associated with muscle necrosis, and iron deprivation has shown potential therapeutic benefits by decreasing muscle necrosis.^307^ Interestingly, iron levels are significantly increased in the gastrocnemius and tibialis anterior muscles of dystrophin-utrophin knockout mice. Treatment with the iron chelator deferoxamine (DFO) in these mice reduced both superoxide levels and dystrophic pathology.^306^ While dietary iron overload did not exacerbate the dystrophic pathology, it did increase total muscle iron content and ferritin expression.^306^ Although no studies to date have directly linked ferroptosis to the pathological progression of DMD, the above-mentioned findings at least suggest that altered iron metabolism is closely associated with DMD and may serve as a viable new target for clinical treatment. Further studies are clearly needed to elucidate the roles of disrupted iron homeostasis and ferroptosis in DMD.

#### Ferroptosis in malignant tumor-induced muscle atrophy

Cachexia—also known as “wasting syndrome”—is a devastating complication that frequently occurs in cancer patients and is associated with a poor prognosis and reduced life expectancy.^308^ Recent studies have shown that SLC39A14 is significantly upregulated in cachectic muscles, both in mouse models and in patients with metastatic cancer.^309^ This upregulation of SLC39A14 inhibits the expression of the key myogenic factors myoblast determination protein 1 (MyoD) and myocyte-specific enhancer factor 2C (MEF2C), blocking the differentiation of muscle cells.^309^ Tandem mass tag analysis of patients with gastric cancer, both with or without sarcopenia, has revealed that ferritin, iron, and oxidative stress may be related to skeletal muscle consumption.^310^ Specifically, gastric cancer patients with sarcopenia exhibit increased muscle oxidative stress and a weakened antioxidant stress system. These patients also show elevated muscle iron content and ferritin expression, regulated by the hepcidin-FPN axis,^310^ suggesting a potential link between ferroptosis and muscle loss in cancer cachexia. Moreover, patients with malignant tumors who have elevated ferritin levels are more likely to develop muscle atrophy.^310^ In addition to its effects on cancer cells, chemotherapy can also cause skeletal muscle atrophy and significantly impair muscle function. For example, chemotherapy agents such as cisplatin are known to induce muscle atrophy during cancer treatment.^311^ Studies in *Pd-1* (programmed cell death protein 1) knockout mice have shown that after cisplatin treatment, the expression levels of ACSL4, HO-1, SAT1, and SLC39A14 are significantly increased in the gastrocnemius muscle,^312^ indicating that ferroptosis plays a role in chemotherapy-induced skeletal muscle atrophy.

#### Ferroptosis in secondary sarcopenia

In addition to age-related primary sarcopenia, chronic diseases, metabolic disorders, or medications can also cause secondary sarcopenia. The increase in global environmental temperatures over the past decades has had a significant impact on human health. Chronic heat exposure causes a reduction in the volume of skeletal muscle, resulting in decreased muscle strength and function in mice.^313^ Results of blood metabolomics have shown that hyperthermia-induced sarcopenia is associated with elevated levels of homocitrulline in serum.^313^ Homocitrulline causes mitochondrial dysfunction in muscle cells by inducing ferroptosis, and supplementation with Nrf2 activators such as Oltipraz can relieve muscle atrophy and dysfunction caused by heat exposure.^313^

### Ferroptosis in myocardial diseases and disorders

#### Ferroptosis triggers cardiomyopathy

Maintaining iron homeostasis is essential for proper cellular function, particularly in cardiac cells. Both iron deficiency and iron overload are associated with cardiomyopathy and heart failure via complex mechanisms.^314^ Knocking out ferritin in mice results in early embryonic death, possibly due to heart failure caused by the excessive deposition of biologically active iron in the myocardium.^315^ Cardiomyocyte-specific *Fth* knockout mice (*Fth*^*MCK/MCK*^ mice) exhibit increased oxidative stress in cardiomyocytes, leading to mild cardiac damage upon aging.^37^ Furthermore, when these mice are fed an iron-rich diet, they suffer severe cardiac injury and hypertrophic cardiomyopathy, displaying molecular characteristics typical of ferroptosis, such as decreased myocardial SLC7A11, GSH, and GPX4 expression, elevated MDA and PTGS2 levels, and increased oxidation of PUFAs.^37^ This suggests that FTH can prevent iron-induced cytotoxicity and cardiomyopathy. Moreover, treatment with Fer-1 reverses the aforementioned molecular features of ferroptosis, reduces cardiac fibrosis and hypertrophy, and mitigates mitochondrial damage in cardiomyocytes, which shows similar results obtained by overexpressing SLC7A11.^37^ These findings indicate that the loss of ferritin in cardiomyocytes leads to heart injury and increases susceptibility to iron overload-related ferroptosis and cardiomyopathy. Enhancing antioxidant capacity may therefore serve as a new therapeutic strategy for preventing and/or treating iron overload-induced cardiomyopathy.

Mitochondrial cardiomyopathy, primarily caused by mitochondrial dysfunction, leads to myocardial ischemia.^316^ The protease OMA1 (metalloendopeptidase OMA1, mitochondrial), expressed in the inner mitochondrial membrane, is activated by mitochondrial dysfunction. Recently, Ahola and colleagues found that the OMA1-DAP3-binding cell death enhancer 1 (Dele1)-ATF4-mediated integration stress response (ISR) is activated in mice with mitochondrial cardiopathy, specifically in cardiomyocyte-specific *Cox10* (protoheme IX farnesyltransferase, mitochondrial) knockout mice. Inhibition of the ISR in these mice affects cardiac GSH metabolism, reduces GPX4 level, and promotes lipid peroxidation in the heart, ultimately leading to ferroptosis.^317^ These results suggest that the OMA1-Dele1-ATF4‒mediated ISR signal transduction pathway may protect against ferroptosis and may delay the progression of cardiomyopathy induced by the absence of cardiac Cox10. Knocking out *Slc7a11* in mice exacerbates cardiomyocyte hypertrophy, increases myocardial PTGS2, MDA, and ROS levels, and worsens Ang II-mediated cardiac hypertrophy and dysfunction, all of which can be prevented by overexpressing SLC7A11.^318^ Interestingly, treatment with Fer-1 in *Slc7a11* knockout mice inhibits cardiomyocyte hypertrophy.^318^ These findings suggest that SLC7A11 may alleviate Ang II-induced myocardial hypertrophy by inhibiting ferroptosis and upregulating SLC7A11 could be a novel therapeutic approach for treating cardiac hypertrophic diseases.

#### Ferroptosis promotes chemotherapy/radiation-induced cardiomyopathy

Ferritin, a spherical heteropolymer composed of light chain (FTL) and heavy chain (FTH) subunits, plays a key role in iron metabolism by storing excess intracellular iron.^319^ The ratio between FTH and FTL varies by tissue type; FTL-rich ferritin is prevalent in iron-storing organs such as the spleen and liver, while FTH-rich ferritin is more abundant in organs with slightly lower iron content, such as the heart.^320^ FTH possesses ferroxidase activity, catalyzing the conversion of Fe^2+^ in the cytoplasm to ferric iron (Fe^3+^), which is then stored in ferritin nanocages. This iron-scavenging function of ferritin reduces intracellular ferrous iron content, thereby preventing the iron-mediated production of ROS and protecting tissues from oxidative damage.^321,322^ Myocardium-specific *Fth* knockout mice display reduced cardiac iron levels and increased oxidative stress, resulting in mild cardiac injury. Moreover, when these mice are fed a high-iron diet, they develop severe cardiac damage and hypertrophic cardiomyopathy, characterized by molecular features typical of ferroptosis, such as reduced GSH levels and increased lipid peroxidation. These changes can be rescued by treatment with Fer-1,^37^ confirming the pivotal role of ferroptosis in cardiac pathology. Additionally, SLC7A11 expression is reduced in FTH-deficient cardiomyocytes, and overexpressing SLC7A11 selectively in cardiomyocytes increases GSH levels and prevents cardiac ferroptosis.^37^ DOX also causes increased m6A modification and METTL3 expression in cardiomyocytes, while inhibition of METTL3 alleviates DOX-induced iron accumulation and ferroptosis in cardiomyocytes by promoting m6A modification of TfR1.^323^

Anthracyclines such as DOX and epirubicin are potent anticancer agents, but their clinical applications are severely limited due to the risk of cardiotoxicity,^324^ as exemplified by DOX-induced cardiomyopathy, which is typically associated with a poor prognosis. Although the precise mechanism underlying DOX-induced cardiomyopathy remains unclear, accumulating evidence suggests that iron overload plays a significant role.^36^ Systemic iron accumulation in rats fed a high-iron diet^325,326^ and in knockout mice lacking the homeostatic iron regulator Hfe significantly increases susceptibility to DOX-induced cardiomyopathy. In contrast, mice fed an iron-deficient diet have a reduced risk of DOX-induced cardiomyopathy and increased survival, suggesting that targeting cardiac iron metabolism may be an effective clinical strategy for ameliorating the side effects of anthracycline chemotherapy. Further studies revealed that DOX accumulates in the mitochondria of cultured cardiomyocytes, increasing mitochondrial ROS levels and elevating both cardiomyocyte and mitochondrial iron levels by inhibiting the function of IRP1 and IRP2 (iron regulatory proteins 1 and 2) and reducing the expression of mitochondrial potassium channel ATP-binding subunit (ABCB8).^327^ Moreover, overexpressing ABCB8 was shown to reduce mitochondrial iron and cellular ROS levels, thereby preventing DOX-induced cardiomyopathy.^327^ DOX also upregulates HO-1 via Nrf2 activation, leading to the degradation of heme in the heart and the release of free iron, which accumulates in the mitochondria and triggers lipid peroxidation. This effect can be reversed by knocking out Nrf2 or using HO-1 antagonist zinc protoporphyrin IX, suggesting that free iron released upon heme degradation is a key factor in DOX-induced cardiac injury.^36^ Ultrasound-targeted microbubble destruction-assisted siHO-1-encapsulated exosomes greatly promotes the release efficiency of siHO-1 in the heart, effectively blocks ferroptosis and DOX-caused cardiactoxicity.^328^ However, the role of HO-1 in myocardial toxicity induced by DOX remains controversial. Wang et al. found that BACH1 expression was significantly upregulated in heart tissues of DOX-treated mice, and Bach1 may promote oxidative stress and ferroptosis by inhibiting the expression of HO-1.^329^ Mitochondrial ferritin is highly expressed in the myocardium, and its deletion in mice increases their susceptibility to DOX-induced cardiac injury, resulting in higher mortality and more severe cardiac morphology changes.^330^ These findings suggest that the cardiotoxic effects of DOX can be attributed to an accumulation of mitochondrial iron, and reducing mitochondrial iron levels may prevent the onset of DOX-induced cardiomyopathy.

GPX4 utilizes GSH to scavenge the harmful byproducts of iron-dependent lipid peroxidation, thereby protecting cells from ferroptosis. Mitsugumin-53 (MG53) protein, as a component of the cell membrane repair system, has cardioprotective effects. MG53 overexpression can increase SLC7A11 and GPX4 levels through promoting p53 degradation, then effectively reduce cardiac ferroptosis and improve DOX-induced cardiotoxicity.^331^ Hydrogen sulfide (H_2_S) is an important gaseous medium in the cardiovascular system. DOX can inhibit the synthesis of endogenous H_2_S, and cardiac-specific *Cse* (encoding cystathionine γ-lyase) knockout significantly aggravates DOX-induced ferroptosis and cardiac dysfunction by eliminating the synthesis of endogenous H_2_S in mice.^332^ On the contrary, H_2_S can promote nuclear translocation of Nrf2 and activate SLC7A11/GSH/GPX4 antioxidant pathway, thereby alleviating DOX-induced ferroptosis and cardiac injury in mice.^332^ In a mouse model of DOX-induced cardiomyopathy, both cytosolic and mitochondrial GPX4 levels were significantly reduced in the myocardium, whereas mitochondrial acrolein (a marker of oxidative damage) and MDA levels were elevated, along with increased myocardial lipid peroxidation and other putative markers of ferroptosis.^224^ These changes were mitigated in GPX4 transgenic mice but exacerbated in heterozygous GPX4-deficient mice, indicating that GPX4 serves as a critical regulator of the progression of DOX-induced cardiomyopathy and implicating ferroptosis may play an important role in this process. The study also demonstrated that overexpressing GPX4 in mitochondria or selectively chelating mitochondrial iron could prevent DOX-induced ferroptosis in cardiomyocytes. DOX promotes excessive lipid peroxidation through the formation of DOX-Fe^2+^ complexes in mitochondria,^224^ leading to mitochondria-dependent ferroptosis. In addition, the use of ferroptosis inhibitor Fer-1 prevented the DOX-induced ferroptosis of cardiomyocytes.^224^ Thus, these findings highlight that mitochondrial-dependent ferroptosis plays a key role in the progression of DOX-induced cardiomyopathy and that ferroptosis is the predominant form of regulated cell death underlying DOX-induced cardiotoxicity.

Radiation-induced heart disease is one of the most serious complications in patients with thoracic radiotherapy. Radiation can inhibit the expression of SLC7A11 and GPX4 in a time- and dose-dependent manner, and induce ferroptosis in cardiomyocytes, which may be one of the important causes of radiation-induced heart disease.^333^ Abelmoschus manihot (L.) has the potential to treat ischemic heart disease. Extracts from A. manihot (L.) could reverse radiation-induced weight loss and cardiac MDA increase in mice, and increase the GSH/GSSH and NADPH/NADP^+^ ratios to improve cardiomyocyte REDOX disequilibrium-mediated ferroptosis.^333^ These results suggest that ferroptosis induced by REDOX imbalance is an important mechanism of radiation-induced cardiomyocyte injury.

#### Ferroptosis contributes to myocardial ischemia/reperfusion injury

Damaged myocardial tissue is significantly more susceptible to I/R injury compared to healthy myocardium.^334^ Iron metabolism disorders are involved in cardiac I/R injury and subsequent heart failure. Single-cell RNA sequencing (scRNA-seq) results have confirmed the importance of ferroptosis-related genes in myocardial I/R injury,^335^ and evidence suggests that blocking ferroptosis can reduce the severity of cardiac I/R injury.^36^ In a mouse model of myocardial I/R, Fang et al. found that levels of cardiac non-heme iron and ferritin were significantly increased in the I/R group compared to sham-operated controls. Pretreatment with the ferroptosis inhibitors Fer-1 and dexrazoxane markedly reduced the myocardial infarct area, thereby protecting against I/R-induced damage. The study suggests that the protective mechanism may involve the preservation of mitochondrial function.^36^

In an in vitro system, Gao et al. demonstrated that serum deprivation induced ferroptosis in cardiomyocytes, identifying transferrin and the amino acid glutamines as the primary inducers of this process.^35^ The authors also found that inhibiting glutamine hydrolysis reduced myocardial ferroptosis, thereby mitigating myocardial I/R injury.^35^ More recently, Li and colleagues utilized a diabetic rat model of myocardial I/R injury and observed that myocardial cell damage was accompanied by an increase in ACSL4 and a decrease in GPX4.^336^ The study further demonstrated that inhibiting ferroptosis reduced myocardial I/R injury, whereas the ferroptosis inducer erastin exacerbated cardiomyocyte injury,^336^ indicating the pathogenic role of ferroptosis in diabetic myocardial I/R injury.

Clinical studies have shown that several antioxidants can alleviate the symptoms of myocardial infarction, suggesting that excess ROS contribute to the progression of the condition,^337^ although the precise mechanism remains unclear. Tandem mass tag-based quantitative proteomics analysis in a mouse model of myocardial infarction revealed significant downregulation of the GSH metabolic pathway and various ROS pathways, particularly GPX4.^338^ Moreover, pharmacological inhibition of GPX4 and siRNA-mediated *Gpx4* knockdown in H9c2 cells led to the accumulation of lipid peroxides. Downregulation of GPX4 under metabolic stress, such as cysteine deprivation, further contributed to lipid peroxide accumulation, culminating in ferroptosis.^338^ Mitochondrial GSH is essential for the clearance of ROS and the maintenance of mitochondrial homeostasis. Studies have shown that myocardial iron overload induced by myocardial I/R injury is accompanied by a decrease of mitochondrial inner membrane protein MPV17.^339^ Overexpression of MPV17 improves the mitochondrial GSH level by regulating the mitochondrial SLC25A10, which significantly reduces the ferroptosis of cardiomyocytes. Further results confirm MPV17 is the downstream protein of Nrf2, and the Nrf2/MPV17/SLC25A10/mitochondrial GSH pathway is involved in the regulation of ferroptosis caused by myocardial I/R injury.^339^

N-acetyltransferase 10 (NAT10) is an RNA acetyltransferase. I/R injury could induce increased NAT10 levels in mouse heart and cardiomyocytes in a p53-dependent manner, then NAT10 is able to induce ac4c modification of mybbp1A, which in turn activated p53 and subsequently inhibits SLC7A11 transcription.^340^ p53 and NAT10 form a positive feedback loop that promotes cardiomyocyte ferroptosis and aggravates myocardiac I/R injury.

#### Ferroptosis is involved in diabetic cardiomyopathy

Myocardial injury is a relatively common complication among patients with diabetes, and the myocardium in diabetics is more sensitive to I/R injury, often resulting in a poorer prognosis.^341–343^ This increased susceptibility is likely due to heightened oxidative stress associated with hyperglycemia and ROS overproduction. The expression level of G protein-coupled receptor containing leucine-rich repeats 6 (LGR6) increased in the heart of diabetic mice, and the overexpression of LGR6 modulates STAT3/PGC1α pathway could reverse disrupted mitochondrial biogenesis and alleviate ferroptosis in cardiomyocytes.^344^ Recombinant R-spondin-3 (RSPO3) treatment to activate LGR6 improves mitochondrial dysfunction, ferroptosis and heart dysfunction in diabetic mice.^344^ Studies have reported that the incidence of myocardial ischemia in diabetic patients is nearly three-fold higher compared to non-diabetic individuals.^345^ In addition, Wang et al. demonstrated that the presence of diabetes or high glucose levels exacerbated myocardial I/R injury in rats by inducing NOX2-related oxidative stress in an AMPK-dependent manner, leading to various forms of programmed cell death, including ferroptosis.^346^ Moreover, Li et al., through both in vitro and in vivo experiments, showed that inhibiting ferroptosis could attenuate diabetic myocardial injury, possibly by mitigating hyperglycemia-induced endoplasmic reticulum stress.^336^ The NCOA4-mediated ferritinophagy is also involved in diabetic cardiomyopathy, and (Pro)renin receptor promotes ferroptosis through activating the NCOA4-mediated ferritinophagy, thereby promoting diabetic cardiomyopathy.^347^

Transient receptor potentiovanilin-1 (TRPV1) is an important member of the transient receptor potential family and a potential target for the treatment of diabetes mellitus and its complications.^348^ Capsaicin, as a TRPV1 agonist, can prevent diabetic myocardial infarction by inhibiting ferroptosis.^349^ Capsaicin therapy significantly improves cardiac function by activating TRPV1 in the heart, promoting Nrf2 nuclear translocation and activating Nrf2/HO-1 signaling pathway, thereby reducing the level of ferroptosis in cardiomyocytes.^349^ Dietary capsaicin may also be a therapeutic strategy to improve myocardial infarction in mice with type 2 diabetes.

### The role of ferroptosis in vascular smooth muscle injury

Tobacco use is a major risk factor for a variety of cardiovascular diseases, including coronary artery disease, stroke, and abdominal aortic aneurysm. Interestingly, previous studies have demonstrated that cigarette smoke extract (CSE) significantly induces cell death in rat VSMCs. This effect was completely prevented by treatment with ferroptosis inhibitors such as Fer-1, liproxstatin-1, and DFO, partially mitigated by the GSH precursor *N*-acetylcysteine (NAC) and the NADPH oxidase inhibitor diphenyleneiodonium chloride, but remained unaffected by inhibitors of apoptosis and necroptosis.^350,351^ Furthermore, VSMCs exposed to CSE exhibited classic features of ferroptosis, including increased *Ptgs2* mRNA, elevated lipid peroxidation, and reduced intracellular GSH,^350^ indicating that ferroptosis may serve as a potential therapeutic target for preventing smoking-induced cardiovascular disease. Mechanistically, the authors identified acrolein and methyl vinyl ketone as the principal inducers of ferroptosis in response to CSE.^350^

Aging of VSMCs contributes to cardiovascular disease by promoting arterial remodeling and stiffness. Promoting ferroptosis signaling can cause VSMC senescence and increase vascular stiffness, and the mechanism may be related to inhibiting nucleo-cytoplasmic shuttle of peroxisome proliferator-activated receptor-γ (PPAR-γ) and activating NCOA4-mediated ferritinophagy,^352^ which has important significance for future efforts to inhibit VSMC ferroptosis and eliminate aging-related cardiovascular diseases.

Vascular calcification is also a significant risk factor for cardiovascular events, commonly observed in hypertension, atherosclerosis, diabetic vascular disease, and others, and is associated with a broad spectrum of adverse events.^353^ VSMCs play an important role in vascular calcification, primarily through their differentiation into osteoblast-like cells, which produce stromal vesicles that deposit calcium phosphate on the vascular wall. Research has shown that iron accelerates the calcification process in cultured human aortic vascular smooth muscle cells (HASMCs), and this process is synergistically increased by the cytokine tumor necrosis factor α (TNF-α). Microarray analysis revealed that IL-24 is upregulated early in the calcification process, and iron induces vascular smooth muscle calcification via IL-24 signaling.^354^ Interestingly, ferroptosis has also been implicated in vascular calcification in patients with chronic kidney disease.^355^ High calcium levels have been shown to induce ferroptosis in rat VSMCs, possibly due to the downregulation of SLC7A11 and a subsequent decrease in GSH content.^356^ Moreover, both erastin-induced GSH depletion and RSL3-mediated inhibition of GPX4 significantly increased ferroptosis and vascular calcification in VSMCs. Conversely, increasing GSH levels with NAC moderated calcification, and inhibition of ferroptosis with Fer-1 decreased mineral deposits in VSMCs and reduced calcification in isolated rat and human arterial rings.^356^ In addition, the expression levels of metal transporters SLC39A14 and SLC39A8 are significantly upregulated during vascular smooth muscle cell calcification.^357^ Overexpression of SLC39A14 and SLC39A8 accelerates VSMCs calcification by promoting intracellular iron accumulation, while inhibition of SLC39A14 and SLC39A8 inhibits ferroptosis, significantly alleviating vascular calcification.^357^

Aortic dissection is an acute, often fatal vascular event characterized by the loss of SMCs, which plays a key role in its pathogenesis. Recent findings by Li et al. showed that key regulators of ferroptosis, such as SLC7A11, FSP1, and GPX4, were downregulated in the aorta of patients with Stanford type A aortic dissection (TAAD). Treatment with the ferroptosis inhibitor liproxstatin-1 significantly reduced the development of β-aminopropionitrile-induced aortic dissection in mice.^358^ Cystine deprivation causes a significant reduction in levels of P300 (E1A-associated 300 kD protein, an endogenous histone acetyltransferase) in HASMCs, while knockdown of *P300* or inhibition of P300 activity can exacerbate cystine deprivation-induced ferroptosis in HASMCs, which may be related to HIF-1α/HO-1 pathway.^359^ Under normal circumstances, P300 and p53 competitively bind HIF-1α to regulate the expression of HO-1, and the decrease in P300 expression facilitates the binding of HIF-1α to p53, thus triggering the overexpression of HO-1 and causing ferroptosis in HASMCs.^359^ In addition, the enzyme METTL3 was found to be significantly upregulated in the aorta of TAAD patients and was inversely correlated with both SLC7A11 and FSP1 levels. More importantly, overexpression of METTL3 in HASMCs promoted erastin- and cystine deprivation-induced ferroptosis, while knocking down METTL3 increased both SLC7A11 and FSP1 expression and reduced ferroptosis,^358^ suggesting that ferroptosis plays a crucial pathogenic role in aortic dissection.

The transition of SMCs from a static “contracting” phenotype to a dedifferentiated and proliferating state is the basis for the development of cardiovascular disease. Neointima formation caused by stent implantation is the main reason for the failure of percutaneous coronary intervention, and ferroptosis has been reported to be positively correlated with neointima formation. RSL3 can aggravate the formation of neointima induced by carotid artery ligation and promote the transformation of VSMC phenotype from contraction phenotype to synthetic phenotype.^360^ Meanwhile, the expression level of ferroptosis-related proteins such as PTGS2 increases. Fer-1 and antioxidant NAC can reverse the effect of RSL3 on the transformation of VSMC phenotype in rats.^360^ Another study also found that ferroptosis stress directly triggered the dedifferentiation of SMCs, and blocking ferroptosis could correct the damaged mitochondrial homeostasis in dedifferentiated SMCs, delaying the phenotypic transformation and arterial remodeling of SMCs.^361^ Mucosa-associated lymphoid tissue lymphoma translocation protein 1 (MALT1) is a type of protein that has proteolytic activity through a caspase-like domain. Pharmacological inhibition of MALT1 triggers ferroptosis in vascular SMCs and helps to improve proliferative vascular disease.^362^ These results indicate that inhibiting ferroptosis may be an attractive strategy to limit vascular restenosis and treat cardiovascular diseases.

**Table 1:** Iron metabolism and ferroptosis in muscle diseases and disorders

Diseases		Iron metabolism	Antioxidant regulation	Lipid metabolism
Skeletal muscle diseases and disorders	Age related-sarcopenia	Iron overload was detected in the atrophic muscles of sarcopenia animal models and patients.^73,291,292^ The expressions of TfR1 decreased and SLC39A14 increased^23,289^	The expression of SLC7A11 was decreased, GSH content was decreased, ROS accumulation and oxidative stress were increased.^73,524^	Adipogenesis‐related genes such as Fasn and Adipoq were significantly induced, and activation of unsaturated fatty acid biosynthesis and lipid peroxidation increased.^23^
	Amyotrophic lateral sclerosis (ALS)	TfR1 and free iron levels were increased,^525^ and the expression of serum ferritin in ALS patients increased.^526^	Hypochlorous acid and its catalytic enzyme MPO increased, and GPX4 expression decreased.^302,303^	C11-BODIPY fluorescence staining showed increased lipid peroxidation.^525^
	Duchenne muscular dystrophy (DMD)	The level of total elemental iron and expression of ferritin and ferroportin increased in muscle.^306^	ROS accumulation and oxidative stress increased.^306^	The production of iron-dependent hydroxyl radicals and lipid peroxidation increased.^307^
	Statin-induced rhabdomyolysis	Atorvastatin increased intracellular iron concentration in cardiomyocytes and muscle satellite cells in a dose-dependent manner.^527^	The cellular and mitochondrial ROS levels of cardiomyocytes and muscle stem cells were significantly increased after atorvastatin treatment.^527^	The expression of proteins related to lipid peroxidation, such as PTGS2/COX-2 and 4-HNE, and MDA levels were increased in cardiomyocytes and muscle stem cells after atorvastatin treatment.^527^
	Glycerol-induced rhabdomyolysis	The expression of ferritin increased.^528^	The level of GSH decreased and the expression of heme oxygenase-1 (HO-1) increased.^528^	The contents of MDA and 4-HNE (lipid peroxidation products) increased.^528^
	Exertional heat stroke (EHS)-induced rhabdomyolysis	Non-heme iron accumulated in muscle tissue. The expression of iron homeostasis related genes in gastrocnemius muscle was dysregulated, such as increased expression of TfR1, NCOA4, SLC39A14, and decreased expression of FTH1.^38^	The expression of GPX4 in gastrocnemius muscle of mice in EHS model group was significantly decreased.^38^	Increased lipid peroxidation, indicated by BODIPY 581/591 fluorescence and the increased lipid metabolites levels of MDA, 5‐HETE, and 15‐HETE were observed in muscle tissue after EHS.^38^ The expressions of ACSL4 and PTGS2 were also significantly increased.^38^
	Malignancy tumor-induced muscular atrophy	The metal ion transporter SLC39A14 was found to be significantly upregulated in cachectic muscles.^309,312^ The iron content and ferritin expression in muscle increased.^310^	ROS, NOX2 and 3-NT levels were significantly elevated.^312^	The levels of ACSL4, HO-1, and 4-HNE were significantly increased.^312^
Myocardial diseases and disorders	Anthracycline-induced cardiomyopathy	HO-1 was significantly upregulated in the hearts of DOX-treated mice and non-heme iron was rapidly and systematically accumulated.^36^	Cellular and mitochondrial ROS levels were increased.^327^ GPX4 expression was decreased, mitochondrial glutathione transporter SLC25A11 was down-regulated, and mitochondrial GSH level was decreased.^529^	The expression of PTGS2 and the levels of MDA and lipid ROS increased.^530^
	Myocardial ischemia/reperfusion injury	The expression of TfR1 and NCOA4 were increased, FTH expression was decreased, and Fe^2+^ was deposited in myocardial tissue.^213,473^	The ROS content of myocardial tissue increased^336^ and the activity of superoxide dismutase (SOD) and expressions of SLC7A11 and GPX4 decreased.^472^	The levels of ACSL4 and MDA increased.^336^
	Diabetic cardiomyopathy	The level of ferritin in myocardial tissue was reduced.^123,185^ Total iron and Fe^2+^ deposits were observed in myocardial tissue and cells.^531^	GPX4 and SLC7A11 levels were reduced in myocardial tissue.^185^ Oxidative stress increased in an AMPK-dependent manner and excess ROS production.^346^	The levels of PTGS2,^123^ ACSL4,^532^ MDA^531^ and 4-HNE^185^ were increased.

*TfR1* transferrin receptor protein 1, *GSH* glutathione, *ROS* reactive oxygen species, *MPO* myeloperoxidase, *GPX4* glutathione peroxidase 4, *PTGS2* prostaglandin-endoperoxide synthase 2, *4-HNE* 4-hydroxynonaldehyde, *MDA* malondialdehyde, *HO-1* heme oxygenase-1, *NCOA4* nuclear receptor coactivator 4, *FTH1* ferritin heavy chain 1, *ACSL4* Acyl-CoA synthetase long-chain family member 4, *NOX2* NADPH oxidase 2, *3-NT* 3-nitrotyrosine, *DOX* doxorubicin, *SOD* superoxide dismutase, *AMPK* adenosine 5’-monophosphate (AMP)-activated protein kinase

## Targeting iron metabolism and ferroptosis to diagnose and treat muscle diseases

Increasing evidence indicates that both iron deposition and ferroptosis are critical mechanisms contributing to muscle diseases and may serve as potential targets to prevent disease progression. In support of this hypothesis, numerous preclinical studies and even clinical trials (Table 2) have demonstrated that ferroptosis inhibitors, such as the iron-chelating agents DFO, DFP, and deferasirox (DFX), have shown promising results.^363–374^ In addition, antioxidants that target ferroptosis—including NAC, CoQ10, and epicatechin—have also exhibited beneficial effects in the clinic.^375–381^ Although current evidence supporting the use of these compounds in treating muscle diseases is not yet overwhelming, their considerable therapeutic potential justifies further investigation.

**Table 2:** Clinic trials targeting ferroptosis in muscle diseases and disorders

Agents		Trial phase and identifier number	Number of participants, and duration	Condition or diseases	Key results	Ref(s)
Iron chelating agents	Combined therapy of deferasirox (DFX) and deferoxamine (DFO)	Phase 2, NCT00901199	22 participants, 12 months	Beta-thalassemia major	Concentration of plasma ferritin, hepatic iron and myocardial iron reduced, and plasma non-transferrin binding iron and unstable plasma iron also decreased.	^363^
	DFX, DFO	Phase 3, NCT00061750	595 participants, 12 months	Beta-thalassemia major	Liver iron concentration decreased significantly in both treatments, and possible dose-related adverse effects, such as elevated serum creatinine levels, should also be considered at high doses of DFX.	^364^
	Combined therapy of DFX and DFO	Phase 2, NCT01254227	60 participants, 24 months	Cardiac iron overload	Iron levels in the liver and myocardium were significantly reduced, and heart function was improved and the incidence of heart failure was reduced. The combined therapy has controllable safety in clinical practice and can rapidly reduce the liver iron content in patients with severe systemic iron burden.	^365^
	DFX, combined therapy of DFX and DFO	Phase 2, NCT00110617	212 participants, 26 months	Sickle cell disease Iron overload Hemolytic Anemia	The serum ferritin concentration after treatment was significantly decreased, and the efficacy of the combined administration group was better than that of DFX group.	
	DFX, DFO	Phase 2, NCT00600938	197 participants, 12 months	Beta-thalassemia patients with myocardial siderosis	Serum ferritin level and myocardial and hepatic iron overload were substantially improved after treatment with DFX or DFO. Left ventricular ejection fraction improved after treatment and remained stable during clinical trial.	^366,367^
	Deferiprone (DFP), DFO	Phase 3, NCT00350662	95 participants 12 months	Hemochromatosis	Iron chelating agent therapy improves systemic iron overload by promoting iron excretion in urine.	^368^
	DFP, combined therapy of DFP and DFO	Phase 4, NCT00733811	213 participants, 60 months	Beta-thalassemia major	Combined treatment with DFP and DFO showed greater advantage in reducing serum ferritin levels compared with the DFP group. DFP alone or in combination with DFO did not differ significantly in cost, survival or adverse events.	^369^
	DFX	Phase 3, NCT00171210	506 participants, 60 months	Beta-thalassemia Iron overload	The concentration of serum ferritin and liver iron decreased significantly after treatment, which could effectively reduce the iron load treatment, and the patients were well tolerated within 5 years of treatment.	^370^
	DFX	Phase 2, NCT00447694	30 participants, 18 months	Beta-thalassemia Iron overload	The risk of abnormally unstable plasma iron and cardiac reactions increases with increasing initial hepatic iron concentration. Monotherapy was effective in patients with mild to moderate iron storage, but did not remove cardiac iron in patients with severe hepatic iron load.	^371,372^
	DFP, DFX	Phase 3, NCT01825512	435 participants, 12 months	Chronic iron overload	During treatment, DFP effectively reduced serum ferritin concentration and cardiac iron concentration, and safely controlled iron overload.	^373,374^
Antioxidants	N-acetylcysteine	Phase not applicable, NCT01778309	20 participants, 2 months	Skeletal muscle damage	N-acetylcysteine supplement could enhance the content of GSH in skeletal muscle.	^375^
	N-acetylcysteine	Phase 1, NCT01537926	42 participants, 12 months	Hypertrophic cardiomyopathy	N-acetylcysteine treatment had little effect on cardiac hypertrophy or fibrosis markers.	^376^
	N-acetylcysteine	Phase 4, NCT01501110	83 participants, 48 hours	Acute myocardial infarction	N-acetylcysteine increased intracellular cysteine and the level of GSH, thereby improving oxidative balance disorder in patients with acute myocardial infarction.	^377^
	Coenzyme Q10	Phase 2, NCT00243932	185 participants, 9 months	Amyotrophic lateral sclerosis	High dose Coenzyme Q10 group has clinical benefit.	
	Coenzyme Q10	Phase 4, NCT02115581	38 participants, 6 months	Dilated cardiomyopathy	Abnormal left ventricular filling and left ventricular ejection fraction were improved in the treatment group.	
	Coenzyme Q10	Phase 2, NCT03133793	216 participants, 3 months	Diastolic heart failure	Treatment significantly improved Kansas City Cardiomyopathy Questionnaire clinical scores, ejection fraction and cardiac function.	^378,379^
	CardioFlex Q10	Phase not Applicable, NCT03826914	69 participants, 3 months	Cardiovascular diseases	CardioFlex Q10 increased heart rate variability and reversed oxidation.	
	Idebenone (a synthetic analog of coenzyme Q10)	Phase 2, NCT00654784	21 participants, 12 months	Duchenne muscular dystrophy	Treatment can significantly improve myocardial degeneration, atrophy, and fibrosis in patients.	
	Idebenone	Phase 3, NCT01027884	65 participants, 12 months	Duchenne muscular dystrophy	Idebenone improved respiratory muscle function and reduced loss of respiratory function, and was safe and well tolerated.	^380^
	(-)-epicatechin	Phase 1, Phase 2, NCT01856868	7 participants, 2 months	Becker muscular dystrophy	Muscle biopsy after treatment showed increased expression of myogenin, transcriptional coactivator gene peroxisome proliferator-activated receptor γ-coactivator 1-α (PGC1α) and AMPK, and decreased expression of myostatin.	

*DFX* deferasirox, *DFO* deferoxamine, *DFP* deferiprone, *GSH* glutathione

### Clinical diagnosis and treatment potential of targeting ferroptosis in sarcopenia

Ferroptosis has emerged as a central focus in research aimed at translating current knowledge into clinically applicable strategies for treating ferroptosis-related diseases.^382^ For example, serum ferritin levels which reflect the body’s iron storage state have been commonly used clinically to evaluate iron load. Several clinical studies have shown a significant correlation between iron accumulation and sarcopenia, with serum ferritin levels being relatively higher in individuals with sarcopenia compared to healthy controls.^383–385^ Moreover, high serum ferritin levels are associated with an increased risk of osteoporosis, sarcopenia, and/or obesity, particularly in women over the age of 50.^386^ Notably, the prevalence of sarcopenia increases with rising serum ferritin levels, showing an odds ratio of 1.74 for sarcopenia in women with the highest serum ferritin levels in comparison to those with the lowest levels.^383^ Another clinical study found that both transferrin saturation and serum ferritin levels were inversely correlated with upper limb muscle strength and muscle mass,^387^ suggesting that iron overload may have a detrimental effect on skeletal muscle tissue. Similar findings were reported in a recent general clinical survey of elderly people in a multinational community.^388^ Notably, another study found that pre-dialysis resistance training increased iron availability and reduced serum ferritin and hepcidin levels in patients with sarcopenia, thereby improving their symptoms.^389^

The above-mentioned clinical studies indicate that the occurrence of sarcopenia may be related to increased serum ferritin levels. However, it is important to note that serum ferritin levels can be influenced by various factors, such as inflammation and trauma,^390^ and older patients tend to present with multiple comorbidities, complicating the diagnose iron deficiency and/or iron overload using serum ferritin levels and transferrin saturation alone. In addition, these clinical studies focused on ferritin levels and transferrin saturation, but did not examine other indicators of iron metabolism, leaving the question of whether iron accumulation is the underlying cause of sarcopenia unsolved. To address this, future clinical studies should include additional markers of serum iron metabolism, such as serum soluble transferrin receptor levels, to better evaluate the putative value of measuring iron metabolism and/or ferroptosis in diagnosing or predicting sarcopenia.

Ferroptosis inhibitors have been used to slow—or even reverse—the progression of muscle disorders.^391^ For instance, iron chelators such as DFO have been identified to reduce ferroptosis in skeletal muscle cells,^392^ while ferrostatins function as free radical-trapping antioxidants to inhibit ferroptosis-related lipid peroxidation.^393^ However, the relatively short biological half-life of DFO limits its clinical applications, highlighting the need for longer-acting, biocompatible ferroptosis inhibitors.^394^ Advances in nanoscale materials have the potential to enhance the safety and efficiency of these drugs, and there is a growing emphasis on screening synthetic ferroptosis inhibitors to reduce ferroptosis and restore skeletal muscle homeostasis. Furthermore, these pharmacological agents can be encapsulated in exosomes, which offer low immunogenicity and high biocompatibility for the delivery of certain compounds.^395^

Overall, although only a limited number of clinical trials have explored the benefits of inhibiting ferroptosis in sarcopenia, the existing evidence indicates that this approach holds promise as an effective therapeutic strategy.

### The prognostic potential of targeting ferroptosis in ALS

There is currently no effective treatment for ALS, and the lack of reliable clinical markers for detecting ALS is one of the principal obstacles hindering the development of new therapies. Bioinformatics analyses of the Gene Expression Omnibus (GEO) datasets GSE153960 and GSE112680 have highlighted significant differences in the expression of ferroptosis-related genes—such as *cytochrome b beta (CYBB)*, *lysosome-associated membrane glycoprotein 2* (*LAMP2)*, *ACSL4*, and *SLC38A1*—between ALS patients and control groups.^300^ Moreover, gene set enrichment analysis (GSEA) revealed that the ferroptosis pathway is more activated in ALS patients, as indicated by higher enrichment score. Notably, elevated expression levels of *CHMP5* and *SLC38A1* in whole blood were associated with a shorter life expectancy in patients.^300^ These findings provide insights into potential new diagnostic and/or prognostic biomarkers for ALS. Interestingly, a randomized, double-blind, phase III trial identified markers of ferroptosis, including ferritin, as being associated with clinical decline in ALS. This association was accompanied by increased levels of 8-oxo-dG (8-oxo-2’-deoxyguanosine, a downstream product of ferroptosis) and lipid peroxidation.^396^ The predictive value of these markers may therefore be helpful for improving patient stratification, guiding individualized care, and allocating resources.

Elevated iron levels in the cerebrospinal fluid (CSF) and serum ferritin levels in patients with sporadic ALS have also been linked to reduced life expectancy.^397,398^ Moreover, iron chelation has been shown to be highly effective at protecting against disease progression in animal models of ALS.^302,399^ For example, the iron-chelating radical scavenger VAR10303 improved motor performance in SOD1^G93A^ transgenic mice, reducing the accumulation of iron and the loss of motor neurons in the spinal cord, ultimately prolonging survival. VAR10303 treatment also attenuated denervation at the neuromuscular junction, decreased the expression of muscle atrophy-related genes in the gastrocnemius muscle, and delayed muscle atrophy.^399^ Similarly, treating SOD1^G86R^ transgenic mice with the iron-chelating agent DFP increased survival compared to placebo-treated mice.^400^ Importantly, clinical trials have demonstrated that DFP treatment reduced iron levels in the motor cortex, medulla oblongata, and spinal cord of ALS patients. DFP-treated patients also exhibited lower levels of oxidative stress-related markers and neurofilament light chains in the CSF.^400^ These results suggest that moderate iron chelation can reduce overall iron levels, possibly providing a new treatment strategy for ALS. Both iron chelation and anti-ferroptosis therapies hold significant potential for treating a wide range of neurodegenerative diseases, including ALS. However, these studies require validation in larger cohorts to fully assess the predictive value of the mentioned biomarkers.

### Potential approaches for the prediction and intervention of ferroptosis in myocardial injury

Both iron deficiency and iron overload are common in myocardial dysfunction and can lead to worse outcomes.^87,401^ Therefore, both low and high ferritin levels are independently associated with the extent of myocardial damage. Importantly, however, studies have failed to demonstrate a role for serum ferritin in predicting myocardial iron content and the occurrence of cardiac complications, as the relationship between serum ferritin and systemic iron status weakens with higher levels of iron load.^402,403^ Currently, cardiac magnetic resonance imaging (CMR) is the most sensitive and consistent non-invasive tool for assessing iron content.^404,405^ Patients with thalassemia—a common genetic disorder characterized by insufficient hemoglobin production—require repeated blood transfusions for many years, often resulting in iron overload that can eventually develop into iron overload-induced cardiomyopathy. CMR data have shown that thalassemia patients with myocardial damage burden a significantly higher risk of cardiac iron overload.^406^ Moreover, the severity of cardiac iron overload, which is correlated with significantly worse clinical outcome, is associated with an extremely increased risk of fatal arrhythmias and/or heart failure.^314,407^ Clinically, the most common strategy to reduce cardiac iron load is intravenous infusions of high-dose DFO, often supplemented by oral DFP.^408^

In addition to its diagnostic value, CMR has important therapeutic applications. For example, CMR can be used to regularly monitor myocardial iron load in transfusion-dependent thalassemia patients receiving iron chelation therapy, thereby reducing cardiac iron overload before overt cardiac dysfunction occurs. Evidence from a randomized controlled trial indicated that DFP is more efficacious than DFO, and combining DFO and DFP is superior to DFO alone.^409^ Furthermore, a new form of CMR, known as multislice multi-echo T2* CMR, has been used to evaluate the distribution of iron in the myocardium.^410^ Results obtained from this segmentation method have shown that iron deposition primarily occurs in the inferior and anterior regions of the left ventricle.^411^ A more recent study demonstrated that R2* CMR can be recruited to noninvasively measure myocardial iron, with more iron observed in the anterior region, followed by the inferior region,^412^ thus confirming the heterogeneity of cardiac iron distribution measured by segmental T2* CMR. The above approaches could be valuable for identifying early iron deposits and/or tracking iron deposition during treatment, thereby helping customize chelation therapy and preventing myocardial dysfunction in clinical practice, as well as reducing myocardial damage caused by ferroptosis.

Altered iron metabolism is a driving factor of ferroptosis, making the use of iron chelators to regulate iron metabolism clinically valuable in treating ferroptosis-related myocardial injury.^6,413^ For instance, DFO has been shown to reduce myocardial I/R injury by blocking ferroptosis.^414,415^ DFP is the first clinically approved oral iron-chelating agent, and a large body of evidence shows that DFP has beneficial effects on the heart, including increased cardiac iron clearance, relief of cardiac symptoms, and prevention of cardiac complications.^416–418^ As monotherapy, DFP is the most cost-efficient therapy, followed by DFX and DFO.^419^ Combination therapies have shown higher efficacy than monotherapies; for example, the DFX/DFO combination is highly effective at reducing serum ferritin and liver iron concentrations, while the DFP/DFO combination better reduces the risk of adverse events.^420–422^ A clinical trial is currently underway to study the effects of SP-420, a novel triterpene oral iron chelator, on myocardial iron overload in patients with β-thalassemia.^423^ Dexrazoxane is also a potent iron chelator that has been shown to chelate free iron ions, extract iron ions from anthracycline metal complexes, prevent free radical production, and block lipid peroxidation.^36^ Notably, unlike other iron chelators, dexrazoxane can directly enter the mitochondria and reduce mitochondrial iron accumulation,^327^ which may explain why other iron chelators are less effective against DOX-induced cardiomyopathy. In addition, a new mitochondria-specific iron chelator called Mito-FerroGreen has shown cardioprotective effects in DOX-treated mice,^224^ further highlighting the importance of chelating mitochondrial iron to protect against myocardial injury.^6^ Despite these benefits, however, it is worth noting that iron chelation therapy is associated with a variety of adverse effects, including non-specific gastrointestinal symptoms and agranulocytosis. Thus, more clinical studies are clearly needed in order to determine the relative efficacy, safety, and long-term clinical benefits of iron-chelating agents.

As one of the first ferroptosis inhibitors identified to inhibit lipid peroxidation, the therapeutic potential of Fer-1 across various diseases has been widely reported. For example, Fer-1 has been shown to alleviate DOX-induced cardiac injury in mice, without affecting iron levels.^36,224^ In addition, Fer-1 has improved cardiac function in animal models of acute and chronic myocardial I/R injury, as well as diabetic cardiomyopathy.^336^ Notably, administration of Fer-1 in vivo is less effective than in vitro applications due to its lower stability in plasma.^424^ UAMC-3203, a novel and highly potent ferroptosis inhibitor derived from Fer-1,^424^ was recently shown to improve myocardial dysfunction following I/R injury.^414^ UAMC-3203 also demonstrated superior to Fer-1 in reducing iron overload-induced lipid peroxidation and multiple organ dysfunction,^425^ suggesting UAMC-3203—or newer derivatives—may be viable candidates for clinical inhibition of ferroptosis. As mentioned previously, the spiroquinoxaline derivative liproxstatin-1, another potent inhibitor of ferroptosis, was shown to protect against myocardial I/R injury in mice, maintaining mitochondrial structural integrity and function by reducing voltage-dependent anion channel 1 (VDAC1) levels and increasing GPX4 levels, thereby diminishing myocardial infarct size.^426^ Collectively, these findings provide compelling evidence supporting the potential of targeting mitochondrial lipid peroxidation and cardiac ferroptosis in the treatment of DOX-induced cardiomyopathy.

## Novel ferroptosis targets and candidate interventions for treating muscle diseases

The past decade has seen a major push towards understanding the complex mechanisms that underlie ferroptosis, and how these mechanisms regulate the muscular system in both health and disease, based on the notion that this knowledge will help researchers identify new treatment targets and intervention strategies designed to reduce ferroptosis (Table 3).

### Potential new targets for ferroptosis in muscular diseases

#### YY1 and YY2

Yin Yang 1 (YY1) and Yin Yang 2 (YY2) are highly homologous members of the Yin Yang family, a class of C2H2 transcription factors that can either activate or suppress expression of their target genes.^427^ These transcription factors play important roles in a variety of biological processes such as VSMC proliferation, neointima hyperplasia,^428^ and the self-renewal and differentiation of embryonic stem cells.^428,429^ Previous studies have shown that YY1 is an epigenetic repressor of several muscle genes, as well as muscle-related miRNAs and lncRNAs that are critical for myoblast differentiation into myotubes. Disruption in the regulatory circuits controlled by YY1 leads to abnormal myogenic differentiation, underlying the pathogenesis of several muscle diseases such as rhabdomyosarcoma and Duchenne muscular dystrophy.^430^ Studies have shown that mice lacking YY1 specifically in cells expressing PAX7 (Paired Box 7, a transcription factor involved muscle stem cell proliferation) exhibit impaired diaphragm development, resulting in neonatal death. Moreover, the absence of YY1 in muscle stem cells significantly hampers muscle regeneration induced by acute injury.^431^ YY1 has also been identified as a key regulator of metabolic reprogramming in muscle stem cells via its dual roles in modulating mitochondria and glycolytic pathways.^431^ A recent study by Li et al. found that YY1 and YY2 compete for binding to the same target sequence in the *SLC7A11* promoter, thereby regulating SLC7A11 expression.^432^ Specifically, they found that binding of YY1 to the *SLC7A11* promoter induces gene expression, resulting in increased GSH content and inhibition of ferroptosis. In contrast, binding of YY2 to the *SLC7A11* promoter suppresses gene expression, reducing GSH production and inducing ferroptosis.^432^ During development, the number of *YY2* transcripts in heart tissue is significantly lower than *YY1* levels, possibly providing a mechanism for reduced ferroptosis during this critical period.^433^

#### ENPP2

Along with iron metabolism and antioxidant signaling pathways, lipid metabolism also plays an important role in regulating ferroptosis in cardiomyocytes and in maintaining myocardial homeostasis. Overexpression of ENPP2 (ectonucleotide pyrophosphatase/phosphodiesterase 2, also known as autotaxin), a lipid kinase crucial for generating lysophosphatidic acid, was shown to moderately increase cell migration and proliferation, and significantly inhibit erastin-induced ferroptosis in H9c2 cells. Mechanistically, ENPP2 protects against erastin-induced ferroptosis in cardiomyocytes by regulating the expression of GPX4, ACSL4, and Nrf2, while also enhancing MAPK and AKT signaling.^434^ Thus, targeting lipid metabolism—in particular through modulating ENPP2—may provide new diagnostic and/or therapeutic approaches for myocardial injury.

#### LRP6

Low-density lipoprotein receptor-related protein 6 (LRP6) is involved in the progression of cardiomyopathy, and studies have shown that loss of LRP6 promotes autophagy and reduces insulin resistance.^435,436^ Moreover, the loss of LRP6 has been reported to promote ferroptosis in cardiomyocytes by regulating autophagy, indicating its involvement in ferroptosis.^437^ Mechanistically, the circular RNA circRNA1615 has been identified to regulate LRP6 expression by sponging miR-152-3p, thereby preventing LRP6-mediated autophagy-related ferroptosis in cardiomyocytes.^437^ This study indicates that targeting the circRNA1615 and miR-152-3p/LRP6 molecular axis may serve as a potential therapeutic target for myocardial infarction.

#### USP7

Deubiquitinases are enzymes that remove ubiquitin from ubiquitinated proteins, playing a vital role in regulating the stability, activity, and translocation of target proteins.^438^ These enzymes help maintain the balance between the ubiquitination and deubiquitination, thereby controlling proteins levels within the cell. Recently, Tang et al. showed that the upregulation of p53 and TfR1 in rats following I/R injury was accompanied by increased ferroptosis and upregulated ubiquitin-specific protease 7 (USP7).^439^ Mechanistically, the authors showed that USP7 promotes myocardial ferroptosis in their I/R model by activating the p53/TfR1 pathway, while inhibiting USP7 protected against myocardial I/R injury by reducing ferroptosis.^439^ This novel USP7/p53/TfR1 pathway may therefore represent a new target for treating myocardial ischemia.

#### ELAVL1

The RNA-binding protein ELAVL1 (embryonic lethal abnormal vision-like protein 1) regulates the expression of target genes such as *TNFA* and *VEGFA* (encoding TNF-α and VEGF-A, respectively) by stabilizing their mRNA, and is associated with cell death and oxidative stress processes.^440^ Recently, Chen et al. showed that surgically induced myocardial I/R injury in rats led to ferroptosis and increased ELAVL1 levels. Additionally, the upregulation of ELAVL1 during I/R diminished enzyme function and cellular antioxidant capacity, manifesting as decreased levels of both GSH and GPX4.^441^ Moreover, inhibiting ELAVL1 was found to suppress ferroptosis and myocardial I/R injury, restoring GPX4 levels and cardiomyocyte viability. Similarly, knocking down ELAVL1 reduced ferroptosis, ameliorated I/R injury, and shrunk myocardial infarct area.^441^ Mechanistically, the study revealed that the transcription factor FOXC1 (forkhead box C1) binds to the *ELAVL1* promoter region during I/R, activating its transcription. Knocking down FOXC1 resulted in reduced ELAVL1 expression, consistent with the notion that FOXC1 regulates ELAVL1 during I/R.^441^ Thus, inhibiting ELAVL1-mediated ferroptosis may provide a novel approach to the treatment of myocardial I/R injury.

#### TRIM21

The ubiquitin E3 ligase TRIM21 (tripartite motif-containing protein 21) ubiquitinates the target protein p62, thereby preventing its oligomerization and sequestration of KEAP1 (Kelch-like ECH-associated protein 1) which downregulating the p62-KEAP1-Nrf2 antioxidant pathway.^442^ In mouse heart tissues and the rat cardiomyocyte line H9c2, loss of TRIM21 increased p62-mediated sequestration of KEAP1. In a DOX-induced cardiomyopathy model, knocking down TRIM21 upregulated the p62-KEAP1-Nrf2 antioxidant pathway, alleviating both DOX-induced mitochondrial deformation and elevated lipid peroxidation levels in cardiomyocytes.^443^ This suggests that TRIM21 plays a role in promoting ferroptosis and cardiotoxicity. Given that knocking out TRIM21 protects against DOX-induced ferroptosis, TRIM21 may serve as a viable therapeutic target for reducing chemotherapy-related cardiotoxicity.

#### MITOL/MARCH5

The mitochondrial E3 ubiquitin ligase MITOL (also known as MARCH5) also plays a key role in regulating mitochondrial structure and function. In cultured cardiomyocytes, exposure to DOX leads to a reduction in both MITOL and GPX4, whereas overexpressing MITOL suppresses DOX-induced ferroptosis by maintaining GPX4 levels.^444^ Knocking down MITOL significantly reduces mitochondrial GPX4 and promotes the accumulation of lipid peroxides in the mitochondria. Moreover, the application of GSH or *N*-acetylcysteine was found to increase GPX4 expression and improve cell viability.^444^ These results indicate that MITOL determines the cell fate of cardiomyocytes by regulating ferroptosis and influencing their susceptibility to cardiomyocytes to DOX.

#### METTL14

An increasing body of evidence indicates that altered gene expression due to chemical modifications in noncoding RNAs is critical for cardiomyocyte injury. For instance, Zhuang et al. demonstrated that treating AC16 cells (a human cardiomyocyte cell line) with DOX led to an upregulation of methyltransferase-like 14 (METTL14), which catalyzes the m6A modification of the lncRNA *KCNQ1OT1*, a miR-7-5p sponge.^445^ In addition, the authors elucidated that the RNA-binding protein insulin-like growth factor 2 mRNA-binding protein 1 (IGF2BP1) interacts with *KCNQ1OT1* to increase its stability and strongly inhibit miR-7-5p activity.^445^ Furthermore, the downregulation of miR-7-5p increased expression of its target gene, *TfR1*, thereby promoting the absorption of iron and the production of lipid ROS, driving DOX-induced ferroptosis in AC16 cells. Furthermore, *METTL14* was shown to be a target gene for miR-7-5p.^445^ These results suggest that the METTL14/KCNQ1OT1/miR-7-5p axis forms a positive feedback loop that regulates ferroptosis in cardiomyocytes, and targeting this axis in cardiomyocytes may provide a novel therapeutic approach to mitigate DOX-induced cardiac injury.

#### PRMT4

Recently, Wang et al. reported that the transcriptional regulator PRMT4 (protein arginine methyltransferase 4), which plays a role in regulating oxidative stress and autophagy, was significantly reduced in DOX-treated cardiomyocytes.^280^ Interestingly, it has been found that overexpressing PRMT4 accelerated ferroptosis and worsened DOX-induced cardiomyopathy, whereas inhibiting PRMT4 activity and reducing its expression produced the opposite effect. Mechanistically, the researchers demonstrated that PRMT4 interacts with Nrf2 to drive its methylation, thereby inhibiting its nuclear translocation and *GPX4* transcription. Finally, the authors showed that the reduction of PRMT4 on DOX-induced ferroptosis could be reversed by either activating Nrf2 or administrating Fer-1. These findings suggest that PRMT4 inhibits Nrf2/GPX4 signaling to exacerbate DOX-induced cardiac ferroptosis, targeting PRMT4 may help prevent the development and/or progression of DOX-induced cardiomyopathy.

#### ACOT1

Omega-6-mediated oxidation of phosphatidylethanolamines (PEs) is a key process that induces ferroptosis, and genes involved in the formation of PUFA-CoA may influence this process. Using RNA sequencing (RNA-seq) analysis, Liu et al. showed significant alteration in the biosynthetic metabolic pathways of PUFAs in DOX-treated mouse hearts, with ACOT1 (acyl-CoA thioesterase 1), which catalyzes the reaction of fatty acyl-CoAs to CoA-SH and free fatty acids, identified as one of the hub genes.^446^ The authors then demonstrated that ACOT1 expression was downregulated in myocardial cells, and overexpressing ACOT1 in cardiomyocytes inhibited DOX-induced ferroptosis, while knocking down ACOT1-sensitized cardiomyocytes to DOX-induced ferroptosis both in vitro and in vivo.^446^ Furthermore, they observed that αMHC-ACOT1 transgenic mice (which overexpress ACOT1 in cardiomyocytes) have an altered composition of free fatty acids composition, suggesting that the beneficial effects of ACOT1 against ferroptosis are related to its enzymatic function in regulating lipid metabolism.^446^ These results indicate that ACOT1 may contribute to protecting against DOX-induced cardiotoxicity, with its beneficial effects potentially stemming from its ability to modulate fatty acid composition.

#### The p53-PARK7 signaling axis

Cellular iron homeostasis is regulated post-transcriptionally via the expression of iron-regulatory proteins such as TfR1, ferritin, and FPN, which bind to the iron-responsive element to maintain intracellular iron levels.^447^ The protein PARK7 (Parkinsonism-associated deglycase 7) counteracts iron overload by regulating the transcription of iron-regulatory proteins and blocking mitochondrial iron uptake. Using mass spectrometry, Pan et al. reported that DOX treatment induces the p53-dependent degradation of PARK7, resulting in disrupted iron homeostasis. Moreover, either knocking out p53 or overexpressing PARK7 in cardiomyocytes restored mitochondrial iron-sulfur (Fe-S) cluster activity and iron homeostasis, inhibited ferroptosis, and alleviated DOX-induced cardiac dysfunction.^448^

#### The PGE2/EP1 pathway

Prostaglandins are a class of bioactive metabolites derived from arachidonic acid. Recently, Wang et al. showed that erastin- and DOX-treated cardiomyocytes exhibit increased production of both prostaglandin E2 (PGE2) and its receptor, EP1. Moreover, activating EP1 significantly reduced erastin- and DOX-induced ferroptosis in cardiomyocytes,^449^ while inhibiting EP1 had the opposite effect. The researchers also believed that cardiomyocyte-specific *Ep1* knockout mice are more susceptible to DOX-induced cardiac injury, an effect that was prevented by treatment with Fer-1. Mechanistically, they found that EP1 triggers an increase in intracellular calcium and activates protein kinase C (PKC)/Nrf2 signaling, thereby protecting cardiomyocytes from DOX-induced ferroptosis by upregulating Nrf2-driven genes that encode antioxidant proteins such as GPX4 and SLC7A11.^449^ These findings implicate that activating the PGE2/EP1 pathway may protect cardiomyocytes from DOX-induced ferroptosis via PKC/Nrf2 signaling and could serve as an attractive target for the prevention and/or treatment of DOX-induced cardiomyopathy.

#### CREG1

Cellular repressor of E1A-stimulated genes (CREG1) is widely expressed in all organs of the organism and plays a very important role in maintaining cell stability.^450,451^ DOX can decrease *CREG1* mRNA and protein levels in cardiomyocytes, and the lack of CREG1 in the heart aggravates the cardiotoxicity caused by DOX. Conversely, overexpression of CREG1 inhibits pyruvate dehydrogenase kinase 4 (PDK4) expression by regulating F-box and WD repeat domain containing 7 (FBXW7)-FOXO1 pathway, thereby inhibiting cardiac ferroptosis induced by DOX and alleviating myocardial injury.^452^ Therefore, CREG1 can be used as a potential target for improving DOX-induced cardiotoxicity.

#### HSF1

Previous studies have shown that ferroptosis inhibitors significantly reduce palmitic acid (PA)-induced cell death in both H9c2 cells and primary cardiomyocytes, indicating that PA-induced myocardial injury may involve ferroptosis.^453,454^ Subsequent studies identified HSF1 (heat shock transcription factor 1) as one key player in this process. Overexpression of HSF1 in cardiomyocytes not only reduced PA-induced ferroptosis and lipid peroxidation, but also regulated iron metabolism-related genes such as *Fth1*, *Tfr1*, and *Fpn*, thereby restoring iron homeostasis.^455^ In addition, HSF1 overexpression also prevented the PA-induced decrease in GPX4 protein levels. Furthermore, *Hsf1* knockout mice exhibited more severe PA-induced ferroptosis, characterized by increased *Fpn* and *Fth* mRNA levels and decreased GPX4 and TfR1 expression.^455^ These findings suggest that HSF1 may act to protect against PA-induced ferroptosis in cardiomyocytes by maintaining cellular iron homeostasis and GPX4 expression, highlighting it as a potential therapeutic target for reducing cardiac ferroptosis.

#### The hTBK1-c.978T>A mutation

Previous studies have demonstrated that motor neuron dysfunction in ALS may be linked to the c.978T>A missense mutation in *TBK1* (hTBK1-c.978T>A), which encodes TANK1-binding kinase 1.^456,457^ The hTBK1-c.978T>A mutation significantly reduced the proliferation of NSC-34 cells (a mouse motor neuron-like hybrid cell line), and this effect was prevented by treatment with Fer-1. Moreover, the hTBK1-c.978T>A mutation significantly increased KEAP1 expression and inhibited Nrf2 signaling in NSC-34 cells, and these effects were partially reversed by knocking down p62.^458^ These results suggest that the hTBK1-c.978T>A mutation induces ferroptosis via the KEAP1/Nrf2/p62 signaling pathway, pointing to a possible new target for ALS.

#### Frataxin

The mitochondrial iron-binding protein frataxin is associated with the biosynthesis of iron-sulfur (Fe-S) clusters and mitochondrial function.^459^ Du et al. found that inhibiting frataxin expression significantly increased erastin-induced ferroptosis by accelerating the accumulation of free iron and lipid peroxidation, leading to severe changes in mitochondrial morphology. These findings suggest that frataxin may be a key regulator of iron-mediated death through its role in maintaining iron homeostasis and mitochondrial integrity.^460^ Recently, Zhang et al. reported that frataxin protein levels increased significantly in mice during myocardial ischemia, followed by reduced reperfusion/reoxidation, and then returned to baseline levels. The study also revealed that knocking down frataxin in H9c2 cells increased their sensitivity to RSL3-induced iron-dependent cell death, whereas overexpression of frataxin exhibited oppositely. Additionally, overexpression of frataxin in the mouse heart reduced myocardial I/R injury by regulating iron homeostasis and inhibiting ferroptosis.^461^ Together, these results indicate that frataxin plays a key role in protecting against ferroptosis in cardiomyocytes and may offer new targets and strategies for mitigating myocardial I/R injury.

#### Circular RNA FEACR

Circular RNAs (circRNAs) play an important role in myocardial I/R injury. Recently, Ju et al. demonstrated that overexpression of circRNA FEACR inhibits hypoxia-reoxygenation-induced ferroptosis, thereby improving myocardial infarction. Conversely, suppression of FEACR induces ferroptosis in cardiomyocytes.^462^ The study further revealed that FEACR regulates ferroptosis in cardiomyocytes through the nicotinamide phosphoribosyltransferase (NAMPT)-SIRT1-FOXO1-FTH axis.^462^ These findings suggest that FEACR and/or its downstream signals may serve as new targets for reducing ferroptosis-related myocardial damage.

#### CircRNA Myst4

Pulmonary hypertension is a cardiopulmonary disease with high morbidity and high mortality, characterized by structural vascular remodeling and abnormal vasoconstriction and structural vascular remodeling, both of which are associated with pathological changes in pulmonary artery smooth muscle cells (PASMCs).^463^ The expression of circRNA Myst4 (circMyst4) decreased significantly in hypoxic environment.^464^ circMyst4 can directly bind to DEAD-box helicase 5 (DDX5) in the nucleus to reduce splicing of *Gpx4* pre-mRNA in cytoplasm, meanwhile, circMyst4 inhibits the formation of eukaryotic translation elongation factor 1 alpha 1 (Eef1A1)/ACSL4 complex in cytoplasm, thus alleviating hypoxia and ferroptosis of PASMCs. Therefore, circMyst4 may inhibit PASMCs ferroptosis as a new potential therapeutic target for ameliorating pulmonary hypertension.

### Targeting ferroptosis as a potential intervention strategy for treating muscle diseases

Impaired cardiac function due to sepsis is associated with a high mortality rate. Using a mouse model of cecal ligation and puncture-induced sepsis, Wang et al. reported that the protein levels of GPX4, SOD, and GSH were significantly reduced in the myocardium, while HO-1 expression, TfR1 expression, and iron concentrations were notably increased. Interestingly, they found that the α2-adrenergic receptor agonist dexmedetomidine reversed these changes, reducing ferroptosis by increasing GPX4 expression and decreasing iron concentration, thereby alleviating sepsis-induced cardiomyocyte damage.^465^

The ferroptosis process involves the production of a large number of free radicals. A natural dietary cannabinoid, β-Caryophyllene (BCP), can terminate free radical chain reactions by interacting with molecular oxygen, protecting cardiomyocytes from cellular ferroptosis caused by cysteine deficiency or GPX4 inactivation.^466^ In addition, BCP can maintain mitochondrial morphological integrity and restore mitochondrial function, and oral administration of BCP (50 mg/kg daily) significantly alleviates DOX-induced myocardial atrophy and cardiomyopathy in mice. These results reveal that BCP may act as a natural anti-ferroptosis compound, and pharmacological modifications based on BCP may offer hope for the treatment of heart disease associated with ferroptosis.

Because ferroptosis has been closely linked to myocardial I/R injury, the use of ferroptosis inhibitors is considered a potential new strategy for reducing the severity of myocardial I/R injury. Polydopamine nanoparticles (PDA NPs) were shown to inhibit the accumulation of Fe^2+^ in H9c2 cells and restore mitochondrial function. In vivo, PDA NPs effectively reduce Fe^2+^ deposition and lipid peroxidation in the myocardial tissue of mice subjected to myocardial I/R.^467^ The New Nano-Resuscitation Solution (TPP-MR) significantly reduces cardiac ferroptosis and myocardial injury by reducing ROS and ACSL4 levels and increasing GSH and GPX4 levels.^468^ In addition, the imidazole etomidate was found to inhibit I/R-induced cardiac ferroptosis, evidenced by reductions in GSH activity and GPX4 expression, as well as increases in MDA and ACSL4 levels. The underlying mechanism may involve activation of Nrf2/HO-1 pathway.^469^ Icariin, a biologically active substance extracted from *Epimedii herba*, has also been shown to reduce I/R-induced ferroptosis in cardiomyocytes by activating the Nrf2 signaling pathway.^470–472^ Furthermore, cyanidin-3-glucoside (C3G) has been reported to reduce the infarct area in mice following I/R, and to lower the expression of oxidative stress and ferroptosis-related proteins such as TfR1, as well as the expression of ferritinophagy-related proteins such as NCOA4, Beclin 1, and the LC3II/LC3I ratio.^473^ In H9c2 cells subjected to OGD/R, C3G also reduced oxidative stress, downregulated TfR1 expression and the LC3II/LC3I ratio, and upregulated the expression of FTH and GPX4.^473^ Isoliquiritigenin has been reported to significantly reduce ROS generation of neonatal mouse cardiomyocytes induced by hypoxia/reoxygenation (H/R) and activate Nrf2/HO-1/SLC7A11/GPX4 pathway to reduce oxidative stress, thereby reducing ferroptosis.^474^ Salvia miltiorrhiza Bunge may inhibit ferroptosis of cardiomyocytes and ameliorate myocardial injury by activating Nrf2 pathway.^475^ Salidroside effectively alleviates Fe^2+^ accumulation, REDOX imbalance and mitochondrial function in I/R-induced myocardial injury by activating AMPKα2, thus improving myocardial I/R injury. These findings suggest inhibiting ferroptosis may be beneficial in protecting against myocardial I/R injury.

Ferritin specifically binds to its receptor at the cell surface and enters the cell via endocytosis.^476^ Thus, apoferritin (ApoFn)—i.e., iron-free ferritin—can be used as an ideal drug delivery vehicle to actively target and inhibit ferroptosis in cells expressing high levels of ferritin receptor.^477^ Cyclosporine A (CsA), a neutral cyclic polypeptide isolated from fungi, has protective effects against myocardial injury; however, its poor water solubility and high affinity for plasma proteins restrict its delivery effectiveness to ischemic myocardial cells.^478^ To overcome these limitations, Qian et al. recently developed CsA@ApoFn, a CsA-loaded nanoparticle in which CsA is encapsulated in ApoFn. The authors demonstrated that CsA@ApoFn accumulates in ischemic myocardial cells via TfR1-mediated endocytosis.^479^ Once inside ischemic cardiomyocytes, the CsA molecules in CsA@ApoFn inhibit apoptosis by increasing mitochondrial membrane potential and reducing the level of oxygen free radicals, while the ApoFn proteins inhibit ferroptosis by driving the expression of GPX4 and decreasing the cell’s lipid peroxide content. This synergistic effect between CsA and ApoFn provides a new strategy for designing additional interventions using other ferroptosis inhibitors.^479^

Advanced glycation end products (AGEs) play a major pathogenic role in diabetic cardiomyopathy and have been shown to induce ferroptosis in engineered cardiac tissues (ECTs), as evidenced by elevated levels of PTGS2 and lipid peroxides, along with reduced levels of ferritin and SLC7A11.^123^ In addition, transmission electron microscopy of AGE-treated cardiomyocytes revealed shrunken mitochondria and increased membrane density, a typical morphological feature of ferroptosis.^123^ Moreover, sulforaphane (a phytochemical commonly found in cruciferous vegetables) inhibits the formation of AGEs and was shown to activate Nrf2 via AMPK signaling, upregulate ferritin and SLC7A11 levels, and inhibit cardiac ferroptosis in both AGE-treated ECTs and a mouse model of diabetic cardiomyopathy.^123^ These findings suggest that sulforaphane might be used as a food supplement to prevent ferroptosis and diabetic cardiomyopathy.

The active cation transporter Na^+^/K^+^-ATPase (NKA) is expressed on the plasma membrane, and reduced NKA activity is strongly associated with DOX-induced cardiomyopathy.^480^ Recently, Leng et al. discovered that the NKA α1 isoform interacts with SLC7A11 to form a novel protein complex, and reduced NKA activity in NKAα1 haplo-insufficient mice exacerbates DOX-induced ferroptosis and cardiac dysfunction.^481^ Furthermore, the authors showed that antibodies targeting the DR region of the NKAα1 subunit (DR-Ab) promote the formation of the NKAα1/SLC7A11 complex, stabilizing SLC7A11 at the cell surface and reducing DOX-induced cardiac dysfunction.^481^ These results suggest that DR-Ab may serve as a novel therapeutic strategy to mitigate DOX-induced cardiotoxicity.

Baicalin has good antioxidant activity, can inhibit autophagy and cell ferroptosis by activating SIRT3 signaling pathway, and alleviate diabetic cardiomyopathy. However, the targeting of baicalin needs to be further improved.^482^ Zeng et al. designed the baicalin-peptide supramolecular self-assembled nanofibers that selectively target the angiotensin type II receptor (AT1R), which is upregulated in DOX-damaged cardiocytes.^483^ Compared with systemic administration, AT1R-targeted baicalin administration can produce effective cardiac accumulation, which makes baicalin more effective in inhibiting ferroptosis in myocardium and improving cardiac function.^483^

Several studies have targeted ACSL4 as a potential treatment for ferroptosis-related conditions. For instance, rosiglitazone—a well-studied activator of PPAR-γ (peroxisome proliferator-activated receptor-γ) activator—has been shown to inhibit lipid peroxidation and ferroptosis in smooth muscle cells and lung epithelial cells by suppressing ACSL4 activity.^18,484,485^ Paeonol (2-hydroxy-4-methoxy acetophenone) was recently reported to significantly inhibit ferroptosis in heme-treated neuronal cells by targeting ACSL4^486^ and may therefore be a viable candidate for treating ferroptosis-related conditions. In addition, protein kinase C epsilon (PKCε) phosphorylates the transcription factor STAT3, thereby inducing gene transcription. Interestingly, PKCε was also identified as a direct target of paeonol, which promotes MFN2-mediated mitochondrial fusion through the activation of the PKCε/STAT3 pathway, thereby preventing DOX-induced cardiotoxicity.^487^ Fucoidan inhibits cardiac ferroptosis induced by DOX through the Nrf2/GPX4 pathway and has the potential to improve DOX-induced cardiotoxicity.^488^ Spexin is a neuropeptide with the effects of neurotransmitter/neuroregulator and endocrine factor.^489^ Treatment with Spexin inhibits ferroptosis in cardiomyocytes by reducing iron accumulation and improving abnormal lipid metabolism, and improve DOX-induced cardiotoxicity.^490^ Protosappanin A (PrA) is an anti-inflammatory compound extracted from hematoxylin. PrA can physically bind to ACSL4 and FTH1, inhibit ACSL4 phosphorylation and subsequent phospholipid peroxidation, and also inhibit FTH1 autophagic degradation and subsequent Fe^2+^ release,^491^ therefore, PrA has protective effect on myocardial I/R- and DOX-induced cardiotoxicity and may become an additional therapeutic option.

Nicorandil is an ATP-sensitive potassium channel opener that has been approved for the treatment of various types of angina.^492^ Patients with diabetic cardiomyopathy have cardiac microvascular dysfunction, and nicorandil treatment promotes phosphorylation and mitochondrial translocation of AMPKα1, which further inhibits ACSL4 and ultimately mitochondria-associated ferroptosis.^493^ Therefore, nicorandil therapy improves diabetic cardiomyopathy by alleviating cardiac microvascular damage and remodeling microvascular structure.^493^ In addition, nicorandil also regulates ferroptosis and alleviates septic cardiomyopathy through the toll-like receptor 4/SLC7A11 signaling pathway.^494^ These studies may provide a basis for the addition of new indications for nicorandil. Ferroptosis and macrophage-induced inflammation are the two main key roles in the process of sepsis-induced cardiac injury. Jiang et al. coordinated ceria nanozyme with curcumin to form CeCH NPs. CeCH NPs not only clear ROS, reverse ferroptosis of cardiomyocytes, but also promote polarization of M2 macrophages and reduce inflammation, thus significantly improving the heart damage induced by sepsis and restoring heart function.^495^

Recently, Ma et al. reported that metformin, a classic drug used to treat diabetes, prevents high-fat diet-induced lipid overload, ferroptosis, and calcification in rat VSMCs both in vitro and in vivo.^496^ The study demonstrated that feeding rats a high-fat diet rich in palmitic acid promoted the expression and secretion of the extracellular matrix protein periostin. Notably, this upregulation of periostin reduced the expression of SLC7A11 in VSMCs by inhibiting p53, thereby leading to decreased GSH production and increased susceptibility to ferroptosis. Moreover, metformin supplementation enhanced the antioxidant capacity of VSMCs by activating Nrf2 signaling,^496^ suggesting that targeting periostin in VSMCs may provide a new strategy for preventing and/or treating vascular calcification, with metformin being a potential candidate. Interestingly, Cai et al. recently showed that low doses of metformin can induce acute kidney injury in mice, presumably via ferroptosis, as blocking ferroptosis prevented metformin-induced nephrotoxicity.^497^ Thus, patients receiving metformin should have their renal iron levels monitored regularly, and their renal function should be evaluated in order to minimize the risk of metformin-induced nephrotoxicity.

Histone methylation is a common process for regulating gene expression. The histone methyltransferase inhibitor BRD4770 was shown to protect against cystine deprivation, erastin-induced ferroptosis, and RSL3-induced ferroptosis in SMCs, with effects comparable to Fer-1 at the optimal concentration.^203^ Mechanistically, BRD4770 reduced lipid peroxidation and ferroptosis in SMCs by inhibiting the methylation of H3K9, upregulating the expression of SLC7A11, SLC3A2, GPX4, and FSP1, as well as promoting the activity of several antioxidant systems.^203^ In addition, inflammation accelerates ferroptosis in SMCs, and BRD4770 inhibits the production of pro-inflammatory cytokines, thereby mitigating the effects of inflammation on ferroptosis.^203^ Together, these results implicate that BRD4770 and/or other histone methyltransferase inhibitors may be highly promising candidates for reducing ferroptosis in smooth muscle cells and in aortic dissection, potentially alleviating aortic dilation by inhibiting inflammation, lipid peroxidation, and ferroptosis.

Edaravone, a synthetic antioxidant marketed as Radicava, specifically targets oxidative damage by interacting with lipid free radicals in cells. Recently, edaravone was approved by the US Food and Drug Administration for the treatment of ALS.^498^ Edaravone has been shown to inhibit cystine deprivation-induced ferroptosis in mouse hepatoma cells and to protect against ferroptosis induced by SLC7A11 and GPX4 inhibitors.^499^ Although no studies to date reported the effects of edaravone on ferroptosis in motor neurons in ALS patients or animal models, its antioxidant properties, and its effects on ferroptosis in cell lines suggest that it may help prevent the progression of ALS by inhibiting ferroptosis in motor neurons.

Compound BY1 improves arterial stenosis by enhancing NCOA4-FTH1 interactions and increasing intracellular Fe^2+^, and induces ferroptosis in VSMCs and neointimal hyperplasia.^500^ Zhang et al. designed a variety of BY1 drug delivery routes, including hydrogel-based BY1 delivery, BY1-coated balloon, and BY1-loaded OPN-targeting nanoparticles (TOP@MPDA@BY1) for targeting proliferative VSMCs. The results have shown that TOP@MPDA@BY1 is the most effective of the three delivery routes,^500^ making TOP@MPDA@BY1 potentially an extremely promising candidate for the development of restenosis treatments.

In addition to chemical compounds, extracellular vesicles (EVs) can also regulate ferroptosis in muscles.^501,502^ In mice, experimentally induced acute myocardial infarction increased expression of DMT1 (divalent metal transporter 1) in the myocardium, and the levels of Fe^2+^, MDA, and GSH indicate that cardiomyocytes undergo ferroptosis following hypoxic injury.^503^ Moreover, overexpression of DMT1 exacerbated hypoxia/reperfusion-induced ferroptosis in cardiomyocytes, whereas knocking down DMT1 significantly reduced ferroptosis.^503^ The researchers also revealed that exosomes derived from human umbilical cord blood-derived mesenchymal stem cells (HUCB-MSCs) inhibited ferroptosis and reduced myocardial injury, whereas exosomes obtained after inhibiting miR-23a-3p, which targets *DMT1*, failed to affect ferroptosis or myocardial injury.^503^ Liu et al intramyocardial administrated healthy cardiac muscle-derived extracellular vesicles (CEVs) into myocardium of mice with myocardial I/R injury and found that CEVs treatment effectively maintained mitochondrial homeostasis and inhibited ferroptosis, thereby reducing cardiac injury and significantly improving cardiac function.^504^ The mechanism may be related to ATP5a1 in CEVs, moreover, ATP5a1 overexpressed adipose-derived stem cell (ADSC) derived EVs also has a similar effect.^504^ Macrophage-derived EVs can effectively reduce iron overload of cardiomyocytes caused by hypoxia, and significantly inhibit oxidative stress and ferroptosis caused by excessive iron.^505^ The mechanism may be related to TfR1 on the surface of macrophage-derived EVs, which can be inherited from macrophages to the surface of EVs, giving EVs the ability to bind to transferrin and remove excess iron.^505^ These findings demonstrate the protective and therapeutic potential of stem cell or healthy cell-derived extracellular vesicles in myocardial injury.

Aortic dissection is a disease with high mortality, and extracellular vesicles derived from mesenchymal stem cells offer a promising strategy to combat ferroptosis and restore intra-aortic degeneration. However, the rapid degradation of extracellular vesicles in the circulatory system limits their clinical application.^506^ Using methacrylate gelatin as the substrate material, Wang et al. constructed a controlled release system of exosomes (Gelma-exos) through three-dimensional (3D) printing technology.^507^ Gelma-exos provides sustained release of exosomes derived from mesenchymal stem cells that inhibit ferroptosis in vascular smooth muscle cells and reverse aortic degeneration. Gelma-exos not only provides an alternative method for the treatment of aortic dissection, but also provides a new inspiration for the treatment of ferroptosis-related diseases.

**Table 3:** Potential new targets and candidates for treating muscle diseases and disorder by targeting ferroptosis

Potential target/candidate		Disease or condition	Mechanisms	Ref(s)
Target	YY1 and YY2	Rhabdomyosarcoma, Duchenne muscular dystrophy	YY1 and YY2 could bind to the promoter of *SLC7A11* to regulate the expression level of SLC7A11.	^431,432^
	Autotaxin (ENPP2)	Myocardial injury	ENPP2 regulated the expression of GPX4 and ACSL4, and enhanced MAPK and AKT signaling to protect cardiomyocytes from ferroptosis.	^434^
	Low-density lipoprotein receptor-related protein 6 (LRP6)	Myocardial infarction	LRP6 deletion promoted ferroptosis in cardiomyocytes through regulating autophagy.	^437^
	Ubiquitin-specific protease 7 (USP 7)	Myocardial I/R injury	Inhibition of USP7 activated p53 by inhibiting deubiquitination, and downregulated expression of TfR1, resulting in alleviating ischemia/reperfusion induced-myocardial ferroptosis.	^439^
	Embryonic lethal abnormal vision protein 1 (ELAVL1)	Myocardial I/R injury	Knockdown or pharmacological inhibition of ELAVL1 restored GPX4 levels, suppressed ferroptosis and ameliorated myocardial ischemia/reperfusion.	^441^
	Tripartite motif-containing protein 21 (TRIM21)	DOX-induced cardiomyopathy	TRIM21 negatively regulated the p62/Keap1/Nrf2 pathway by ubiquitinating p62, and knockdown of *Trim21* upregulated the p62/Keap1/Nrf2 pathway, inhibited the mitochondrial deformation and lipid peroxidation level of cardiomyocytes, and alleviated DOX-induced cardiomyopathy	^443^
	MITOL/MARCH5	DOX-induced cardiomyopathy	E3 ubiquitin ligase *Mitol* knockdown significantly reduced mitochondrial-localized GPX4, promoted the accumulation of lipid peroxides in mitochondria and exacerbating DOX-induced ferroptosis.	^444^
	Methyltransferase-like 14 (METTL14)	DOX-induced cardiomyopathy	METTL14 catalyzed m6A modification of the long noncoding RNA *KCNQ1OT1*, a miR-7-5p sponge, led to the increase of miR-7-5p target gene TfR1, promoted iron absorption and lipid peroxide production, and promoted DOX-induced ferroptosis.	^445^
	Protein arginine methyltransferase 4 (PRMT4)	DOX-induced cardiomyopathy	PRMT4 interacted with Nrf2 to promote its enzymatic methylation, thereby inhibiting nuclear translocation of Nrf2 and subsequent GPX4 transcription and accelerating DOX-induced myocardial ferroptosis.	^280^
	Acyl-CoA thioesterase 1 (Acot1)	DOX-induced cardiomyopathy	Acot1 overexpression inhibited DOX-induced cardiomyocyte ferroptosis.	^446^
	Park7	DOX-induced cardiotoxicity	Overexpression of Park7 significantly restored mitochondrial Fe-S cluster activity and iron homeostasis, inhibited ferroptosis, and salvaged DOX-induced cardiac dysfunction.	^448^
	EP-1	DOX-induced cardiotoxicity	EP-1 activated PKC/Nrf2 in cardiomyocytes and protected cardiomyocytes from DOX-induced ferroptosis by promoting Nrf2-driven antioxidant gene expression.	^449^
	CREG1	DOX-induced cardiotoxicity	CREG1 inhibits PDK4 expression by regulating FBXW7/FOXO1 pathway, thereby alleviating DOX- induced myocardial injury through inhibiting cardiac ferroptosis.	^452^
	Heat shock factor 1 (HSF1)	Diabetic cardiomyopathy	HSF1 overexpression in cardiomyocytes improved iron homeostasis by regulating the expression of iron metabolism-related genes (such as FTH1, TfR1, and FPN), inhibited endoplasmic reticulum stress, and up-regulated the expression of GPX4, thereby inhibiting lipid peroxidation and alleviating high fat-induced ferroptosis.	^455^
	*hTBK1-c.978T*>*A mutation*	Amyotrophic lateral sclerosis	hTBK1-c.978 T > A mutation significantly increased Keap1 expression, inhibited Nrf2 signaling, and significantly inhibited neurons activity by inducing ferroptosis.	^458^
	Frataxin	Myocardial I/R injury	Cardio-specific overexpression of frataxin improves myocardial I/R injury by regulating iron homeostasis and inhibiting ferroptosis.	^461^
	*circRNA (FEACR)*	Myocardial I/R injury	*FEACR* regulated cardiomyocyte ferroptosis through NAMPT-SIRT1-FOXO1-FTH1 signal axis to improve myocardial infarction.	^462^
Candidate	Dexmedetomidine (Dex)	Sepsis	Dex reduced ferroptosis by enhancing GPX4 and reducing TfR1 and iron concentrations and attenuated sepsis-induced cardiomyocyte damage.	^465^
	β-Caryophyllene	DOX-induced cardiotoxicity	β-Caryophyllene can terminate free radical chain reactions by interacting with molecular oxygen, protecting cardiomyocytes from cellular ferroptosis.	^466^
	Polydopamine nanoparticles (PDA NPs)	Myocardial I/R-injury	PDA NPs inhibited Fe^2+^ accumulation and restored mitochondrial function, and effectively reduced lipid peroxidation in myocardial tissue.	^467^
	Etomidate	Myocardial I/R injury	Etomidate inhibited ischemia/reperfusion induced cardiac ferroptosis via activating Nrf2 pathway, increasing GSH activity and GPX4 expression, and reducing malondialdehyde and ACSL4 levels.	^469^
	Icariin	Myocardial I/R injury	Icariin attenuated I/R-induced cardiomyocyte ferroptosis by activating the Nrf2 signaling pathway.	^470^
	Naringenin	Myocardial I/R injury	Naringenin attenuated I/R-induced cardiomyocyte ferroptosis by activating the Nrf2 signaling pathway.	^472^
	Astragaloside IV	Myocardial I/R injury	Astragaloside IV attenuated I/R-induced cardiomyocyte ferroptosis by activating the Nrf2 signaling pathway.	^471^
	Cyanidin-3-glucoside (C3G)	Myocardial I/R injury	C3G attenuated the expression of oxidative stress and ferroptosis-related proteins such as TfR1, inhibited the expression of ferritinophagy-related proteins, thereby reduced the infarction area of myocardial I/R mice.	^473^
	Isoliquiritigenin	Myocardial I/R injury	Isoliquiritigenin reduces oxidative stress by activating the Nrf2/HO-1/SLC7A11/GPX4 pathway and alleviates ferroptosis.	^474^
	Salidroside	Myocardial I/R injury	Salidroside improves myocardial I/R injury by activating of AMPKα2 and inhibiting ferroptosis.	^533^
	CsA@ApoFn	Myocardial I/R injury	CsA in CsA@ApoFn inhibited the apoptosis of ischemic cardiomyocytes, while ApoFn inhibited the ferroptosis of ischemic cardiomyocytes by promoting the expression of GPX4 protein and decreasing the content of lipid peroxide.	^479^
	Sulforaphane	Diabetic cardiomyopathy	Sulforaphane activated Nrf2 through AMPK signaling, upregulated ferritin and SLC7A11 levels, and inhibited cardiac ferroptosis.	^123^
	DR-Ab	DOX-induced cardiotoxicity	DR-Ab reduced ferroptosis by promoting the combination of NKA-α1 /SLC7A11 complex and alleviated DOX-induced cardiac dysfunction.	^481^
	Paeonol	DOX-induced cardiomyopathy	Paeonol promoted MFN2-mediated mitochondrial fusion by activating the PKCε-STAT3 pathway, thereby preventing DOX-induced cardiotoxicity	^487^
	Baicalin	DOX-induced cardiomyopathy	Baicalin-peptide supramolecular self-assembled nanofibers can produce effective cardiac accumulation, which makes baicalin more effective in inhibiting ferroptosis in myocardium.	^483^
	Protosappanin A	Myocardial I/R- and DOX-induced cardiomyopathy	Protosappanin A has protective effects on myocardial injury by targeting ACSL4/FTH1 Axis-dependent ferroptosis.	^491^
	Metformin (Met)	Vascular calcification	Met supplementation enhanced the antioxidant capacity of vascular smooth muscle cells by activating Nrf2 signaling.	^496^
	BRD4770	Aortic dissection	BRD4770 attenuated lipid peroxidation and SMCs ferroptosis by inhibiting the methylation of H3K9, upregulating the mRNA levels of SLC7A11, SLC3A2, GPX4 and FSP1, promoting the activity of multiple antioxidant systems and inhibiting the production of pro-inflammatory cytokines.	^316^
	TOP@MPDA@BY1	Vascular restenosis	TOP@MPDA@BY1 can improve arterial stenosis by promoting ferritinophagy to induce ferroptosis in VSMCs and neointima hyperplasia.	^500^
	Human umbilical cord blood-derived MSCs (HUCB-MSCs) exosomes	Acute myocardial infarction	HUCB-MSCs-exosomes inhibited DMT1 expression via miR-23a-3p, thereby inhibiting ferroptosis and attenuating myocardial injury.	^503^
	Healthy cardiac muscle-derived extracellular vesicles (CEVs)	Myocardial I/R injury	ATP5a1, which is rich in CEVs, can maintain mitochondrial homeostasis and inhibit ferroptosis, thereby improving heart damage.	^504^
	Macrophage-derived extracellular vesicles	Myocardial hypoxia injury	Macrophage-derived extracellular vesicles effectively reduce iron overload and significantly inhibit oxidative stress and ferroptosis of cardiomyocytes caused by hypoxia.	^505^
	Gelma-exos	Aortic dissection	Gelma-exos provides sustained release of exosomes derived from mesenchymal stem cells that inhibit ferroptosis in vascular smooth muscle cells and reverse aortic degeneration.	^507^

*HSF1* Heat shock factor 1, *TfR1* transferrin receptor protein 1, FTH1 ferritin heavy chain 1, *FPN* ferroportin, *GPX4* glutathione peroxidase 4, *ENPP2* autotaxin, *ACSL4* acyl-CoA synthetase long-chain family member 4, *Nrf2* NFE2 related factor 2, *MAPK* mitogen-activated protein kinase, *AKT/PKB* protein kinase B, *USP7* ubiquitin-specific protease 7, *USP22* ubiquitin-specific protease *SIRT1* sirtuin-1, *ROS* reactive oxygen species, *TRIM21* tripartite motif-containing protein 21, *METTL14* methyltransferase-like 14, *PRMT4* protein arginine methyltransferase 4, *Acot1* acyl-CoA thioesterase 1, *LRP6* Low-density lipoprotein receptor-related protein 6, *ELAVL1* embryonic lethal abnormal vision protein 1, *YY1* Yin Yang 1, *YY2* Yin Yang 2, *PD-1* programmed cell death protein 1, *HO-1* heme oxygenase-1, *PKC* protein kinase C, *I/R* ischemia/reperfusion, *FOXO1* forkhead box protein O1, *PDK4* pyruvate dehydrogenase kinase 4, *FBXW7* F-box and WD repeat domain containing 7, *Dex* Dexmedetomidine, *PDA* *NPs* polydopamine nanoparticles, *C3G* Cyanidin-3-glucoside, Met Metformin, *FSP1* ferroptosis suppressor protein 1, *STAT3* signal transducer and activator of transcription 3, *TLB* trilobatin, *HUCB-MSCs* human umbilical cord blood‒derived mesenchymal stem cells, *DMT1* divalent metal transporter 1

## Limitations and future perspectives

An overwhelming amount of evidence emphasizes that ferroptosis is essential for development and progression of various forms of muscle disease. However, many questions remain to be addressed before ferroptosis can be fully leveraged for clinical diagnostics and therapeutic evaluation. Recent technological advances have proven invaluable in detecting ferroptosis and developing ferroptosis-targeted therapies. Although the application of these technologies in muscle diseases is currently limited, they have contributed to our understanding of the role ferroptosis plays in these conditions (Table 4). First, while our understanding of ferroptosis increased, demonstrating that a single ferroptosis inhibitor or inducer can affect cell death is not necessarily sufficient for the involvement of ferroptosis in disease pathology. Ferroptosis is characterized by iron accumulation and iron-dependent lipid peroxidation, which are critical findings given that other iron-dependent forms of cell death may contribute to iron-mediated lysosomal toxicity.^508^

**Table 4:** Summary of new technologies for targeting ferroptosis in the treatment of muscle diseases

Novel technology	Mechanism	Application	Ref(s)
Fluorescent probes	DRhFe detects the concentration of Fe^3+^ through the reversible chelation of Fe^3+^ with HEDTA. FIP-1 and COU-LIP-1 detects Fe^2+^ based on fluorescence resonance energy transfer mechanism. Ac-MtFluNox, Lyso-RhoNox and ER-SiRhoNox detect labile Fe^2+^ in mitochondria, lysosomes, and endoplasmic reticulum, respectively. Mito-QL and MBI-OMe detect mitochondrial hypochlorous acid (HOCl) levels, and HP, BT-HP, QS-4, and BTFMB detect H_2_O_2_ levels, while H-V detects hydroxyl radical.	Ferroptosis involves complex physiological and pathological processes, and the levels of many important bioactive substances and physiological microenvironment will change during ferroptosis. The use of fluorescent probes to monitor the fluctuating levels of key targets during ferroptosis is of great significance for the diagnosis and drug development of muscle diseases and disorders.	^86,509–513,534–539^
Aggregation-induced emission probe	The probe TPabtBP is specifically localized to lipid monolayers and can detect the presence of lipid droplets.	The probe TPabtBP can detect the dynamic changes of lipid droplets during myocardial ischemia-/reperfusion (I/R) injury, and can be a potential therapeutic target for early intervention of myocardial I/R injury.	^540^
Photochemical activation of membrane lipid peroxidation (PALP)	PALP uses a high-power pulsed laser to photochemically induce lipid peroxidation of local polyunsaturated fatty acid acyl groups in cells or tissue samples, using BODIPY-C11 as a lipid peroxidation indicator, and fluorescence intensity is captured and detected by a lipid peroxidation sensor.	PALP enables rapid screening and non-invasive assessment of patient tissue sensitivity to ferroptosis and tissue sensitivity to ferroptosis inducers, enabling personalized and effective drug treatment for ferroptosis. PALP can assist the research on the biological function of polyunsaturated lipids and lipid peroxidation.	^514^
Proteolysis-targeting chimeras (PROTACs)	PROTACs are heterobifunctional small molecule compounds containing two different ligands, an E3 ubiquitin ligase ligand a ligand that binds the protein of interest (POI) in target cells. The two ligands are connected by a linker to form a “three-body” polymer the target protein ligand-linker-E3 ligand. After entering cells, the POI ligand can specifically bind to its corresponding target protein, while the other end of the ligand can recruit E3 ligase, which can mediate the ubiquitination of POI by ubiquitin binding enzyme E2 for proteosomal degradation.	PROTACs connects the target protein ligand with the E3 ubiquitin ligand to form a ternary complex through appropriate linking chains, which promotes ubiquitin degradation of ferroptosis-associated proteins, such as SLC39A14 and acyl-CoA synthetase long-chain family member 4, to develop therapies targeting muscle diseases. On that basis, CLIPTAC can improve bioavailability, PhotoPROTAC can spatiotemporal control protein degradation using optical methods, and RNA-PROTAC can degrade non-degradable RNA-binding proteins.	^515–517,541^
Lipid nanoparticles (LNPs)	LNPs can increase the water solubility of hydrophobic drugs, extend the residence time of drugs, and improve the efficiency of controlled drug release.	Combining LNPs with PROTAC can improve the biocompatibility and cell selectivity of PROTAC molecules, and may be valuable in the development of potential therapeutic agents targeting muscle diseases.	^517,542–546^
Photodegradation-targeting chimera (PDTAC)	PDTACs consist of three functional modules, a targeting ligand that binds the POI, a photosensitizer that generates reactive oxygen species upon light irradiation, and a linker that connects the ligand and photosensitizer.	PDTAC is a targeted photodegradation strategy that could specifically degrade ferroptosis-associated proteins.	^520^

Second, studying changes in key molecules during ferroptosis is important for designing new therapies. For example, the ratiometric FRET (fluorescence resonance energy transfer)-based Fe^3+^ probe DRhFe was developed by Gao et al. to measure Fe^3+^ levels,^509^ while the endoperoxide reactivity-based FRET probe FIP-1 can directly detect concentration-dependent changes in Fe^2+^, reflecting changes in the labile iron pool during ferroptosis.^510^ In addition, Hirayama et al. developed a series of organelle-targeted selective probes called Ac-MtFluNox, Lyso-RhoNox, and ER-SiRhoNox to detect labile Fe^2+^ pools in mitochondria, lysosomes, and the endoplasmic reticulum, respectively.^511^ These probes are likely to be extremely helpful in monitoring organelle-specific changes in Fe^2+^ during ferroptosis. Interestingly, H-V, a dual-function fluorescence probe developed by Li et al., can detect hydroxyl radical (·OH) levels.^512^ However, the use of fluorescent probes to measure iron and ROS is still in its infancy, with most studies to date conducted in cell cultures and need to be validated at tissue and in vivo levels. In addition, these probes have several limitations that waiting to be overcome, including their short wavelength, low signal-to-noise ratios, and susceptibility to interference.^511,513^ Therefore, next-generation probes with high specificity and sensitivity are required in order to detect changes during ferroptosis.

Furthermore, small-molecule inducers and inhibitors of ferroptosis are valuable for developing ferroptosis-targeted clinical therapies. However, issues such as specificity, biological relevance, pharmacokinetics, and pharmacodynamics must be considered when using these molecules. Photochemical activation of membrane lipid peroxidation (PALP) is an imaging technique recently developed for the in situ detection and prediction of a tissue’s sensitivity to ferroptosis inducers.^514^ PALP can rapidly identify whether a given ferroptosis modulator is likely to have a potential therapeutic effect in patients in a clinical trial, thereby facilitating the development of new clinical strategies for ferroptosis-related muscle diseases and conditions. Another recent technique is the use of engineered proteolysis-targeting chimeras (PROTACs) to degrade targeted proteins in living cells via the ubiquitin-proteasome pathway.^515^ Importantly, PROTACs exhibits high selectivity for their targets and high tissue specificity.^516^ Luo et al. recently reported that a PROTAC targeting GPX4 (dGPX4) depleted endogenous GPX4 and induced ferroptosis in HT-1080 fibrosarcoma cells, with fivefold higher potency than the conventional GPX4 inhibitor ML162.^517^ Another interesting new approach is lipid nanoparticles (LNPs), a novel type of lipid vesicles containing a homogeneous lipid core, have been widely used to deliver nucleic acids and small-molecule drugs.^518^ Based on this approach, Luo et al. developed a tool called dGPX4@401-TK-12 by encapsulating their dGPX4 PROTAC in ROS-degradable LNPs, achieving targeted drug delivery with higher efficacy and safety.^517^ This strategy of selectively degrading GPX4 via biodegradable LNPs containing PROTACs can be expanded to target other key regulators of ferroptosis, aiding in mechanistic study and the development of new therapeutic agents for muscle diseases.

In addition, when using a pharmacological inhibitor to assess whether an enzyme modulates ferroptosis, it is crucial to ensure the inhibitor specifically and selectively targets the enzyme of interest without affecting other physiological processes. For example, GPX4 lacks a drug-like binding pocket on its surface, and classic GPX4 inhibitors such as RSL3 and ML210 act by covalently binding to the enzyme’s active site.^519^ this mechanism may lead to relatively low selectivity and/or induce systemic toxicity when used in vivo. To overcome this potential limitation, Liu et al. recently developed a targeted photolysis approach called photodegradation-targeting chimeras (PDTACs), demonstrating efficient and highly selective degradation of GPX4 without affecting GPX1, another member of the GPX family.^520^ This PDTAC-based strategy can also be used to degrade proteins that are upregulated during ferroptosis, such as proteins related to lipid metabolism and lipid oxidation, thereby preventing and/or slowing the development of muscle diseases. Moreover, when using small molecules in in vivo, the appropriate formulation and route of administration must be considered in order to achieve an acceptable pharmacokinetics profile and pharmacodynamic response in target tissues. Many small molecules can be potent and selective when used in cellular assays but be less efficacious when used in vivo due to poor solubility, stability, and/or pharmacokinetics. For example, RSL3 has low solubility, making it difficult to measure its pharmacokinetics in mice, and thus it is less suitable for animal studies unless directly injected into the target tissue. Erastin has both low solubility and low metabolic stability, limiting its use for animal studies. Moreover, iron chelators should be used with caution in vivo, as iron depletion can have harmful effects beyond merely inhibiting ferroptosis in the target tissue.

Finally, although ferroptosis is largely distinct from other forms of cell death, recent studies have shown that cross-talk can occur between ferroptosis and other types of cell death such as apoptosis and autophagy.^224,521^ For example, many so-called “core regulators” of ferroptosis such as SLC7A11, GPX4, Nrf2, and p53 have also been implicated in other types of cell death. Moreover, multiple forms of cell death—and their mechanisms—can work together in the pathophysiology of muscle disease. Understanding this cross-talk may provide valuable insights into the pathogenic role of ferroptosis in muscle disease and the development of clinical interventions. However, this cross-talk also adds complexity to research. Elucidating these mechanisms and developing new strategies may be critical to harnessing our knowledge of ferroptosis for therapeutic purposes. In addition, addressing these unanswered yet important questions will shed new light not only on ferroptosis, but also on its increasingly diverse role in health and disease.

As the largest organ system in the human body, muscles are not only involved in body movement and immune function, but they can also exert a wide range of beneficial effects on metabolism, cardiovascular function, and mental health through their autocrine, paracrine, and endocrine properties.^522^ For example, a rising volume of evidence suggests that skeletal muscle is more than just a motor unit, but also secretes over 300 putative myokines, and muscle atrophy and injury not only affect the body’s movement and stability, but also alter the release of these myokines, impacting the entire body’s metabolism.^523^ In recent years, ferroptosis has received widespread attention for its critical role in the pathogenesis and progression of muscle diseases. Although no clinical trials using ferroptosis-specific inhibitors for muscle disease have been reported to date, selectively inhibiting ferroptosis has significantly improved muscle function in various animal models. In this respect, ferroptosis research is extremely timely, and exiting new advances are providing fresh opportunities for developing tissue-specific and/or cell type-specific interventions targeting ferroptosis for a wide range of clinical applications. Future discoveries are likely to push these boundaries even further, leading to the new treatments—and even prevention—of these devastating muscle diseases and disorders.
